# Digging the compromise: investigating the link between limb bone histology and fossoriality in the aardvark (*Orycteropus afer*)

**DOI:** 10.7717/peerj.5216

**Published:** 2018-07-11

**Authors:** Lucas J. Legendre, Jennifer Botha-Brink

**Affiliations:** 1 Karoo Palaeontology Department, National Museum, Bloemfontein, South Africa; 2 Department of Zoology and Entomology, University of the Free State, Bloemfontein, South Africa

**Keywords:** Bone histology, Aardvark, Fossoriality, Mammals, Environmental constraints

## Abstract

Bone microstructure has long been known as a powerful tool to investigate lifestyle-related biomechanical constraints, and many studies have focused on identifying such constraints in the limb bones of aquatic or arboreal mammals in recent years. The limb bone microstructure of fossorial mammals, however, has not been extensively described. Furthermore, so far, studies on this subject have always focused on the bone histology of small burrowers, such as subterranean rodents or true moles. Physiological constraints associated with digging, however, are known to be strongly influenced by body size, and larger burrowers are likely to exhibit a histological profile more conspicuously influenced by fossorial activity. Here, we describe for the first time the limb bone histology of the aardvark (*Orycteropus afer*), the largest extant burrowing mammal. The general pattern is very similar for all six sampled limb bones (i.e., humerus, radius, ulna, femur, tibia, and fibula). Most of the cortex at midshaft is comprised of compacted coarse cancellous bone (CCCB), an endosteal tissue formed in the metaphyses through the compaction of bony trabeculae. Conversely, the periosteal bone is highly resorbed in all sections, and is reduced to a thin outer layer, suggesting a pattern of strong cortical drift. This pattern contrasts with that of most large mammals, in which cortical bone is of mostly periosteal origin, and CCCB, being a very compliant bone tissue type, is usually resorbed or remodeled during ontogeny. The link between histology and muscle attachment sites, as well as the influence of the semi-arid environment and ant-eating habits of the aardvark on its bone microstructure, are discussed. We hypothesize that the unusual histological profile of the aardvark is likely the outcome of physiological constraints due to both extensive digging behavior and strong metabolic restrictions. Adaptations to fossoriality are thus the result of a physiological compromise between limited food availability, an environment with high temperature variability, and the need for biomechanical resistance during digging. These results highlight the difficulties of deciphering all factors potentially involved in bone formation in fossorial mammals. Even though the formation and maintaining of CCCB through ontogeny in the aardvark cannot be unambiguously linked with its fossorial habits, a high amount of CCCB has been observed in the limb bones of other large burrowing mammals. The inclusion of such large burrowers in future histological studies is thus likely to improve our understanding of the functional link between bone growth and fossorial lifestyle in an evolutionary context.

## Introduction

Fossorial activity, that is, the process of digging soil for food or shelter, is associated with many physiological, anatomical, and ecological constraints, which have been documented in all major tetrapod clades (Hildebrand, 1974, 1985). Among Mammalia, in particular, several groups present many morphological adaptations to a fossorial, semifossorial, or subterranean lifestyle that have appeared independently through evolutionary convergence. Such adaptations have been described in a comparative framework since the early 20th century (Shimer, 1903). Osteological and muscular characters linked with fossoriality in extant mammals have been the focus of many morphoanatomical studies in the last few decades, for example, on monotremes (Gambaryan et al., 2015), marsupials (Warburton et al., 2013), xenarthrans (Vizcaíno, Fariña & Mazzetta, 1999; Vizcaíno & Milne, 2002; Olson et al., 2016), rodents (Gasc et al., 1985; Thorington, Darrow & Betts, 1997; Elissamburu & Vizcaíno, 2004; Lagaria & Youlatos, 2006; Samuels & Van Valkenburgh, 2008; Elissamburu & De Santis, 2011), true moles (Rose et al., 2013), golden moles (Gasc et al., 1986), and mustelids (Ercoli et al., 2013, 2015; Moore et al., 2013; Rose et al., 2014). Many of these studies have focused on the forelimb of scratch-diggers, that is, animals that dig by alternately flexing and extending their limbs to cut and loosen the soil with their claws (Hildebrand, 1974), and have identified several anatomical markers linked with fossoriality in the limb bones of scratch-digging mammals (e.g., enlarged medial epicondyle in the humerus, elongated olecranon process in the ulna), which have been used to define quantitative measurements to assess degrees of fossoriality among these groups (see reviews in Hildebrand, 1985; Reichman & Smith, 1990). These measurements reflect strong biomechanical constraints on bone macrostructure and associated muscle attachments; as such, they have also been included in paleontological studies to predict a fossorial lifestyle in extinct mammals from their macroanatomy, most notably in extinct xenarthrans (Bargo et al., 2000; Vizcaíno et al., 2001; Toledo, 2016), along with other methods such as muscular reconstruction (Toledo, Bargo & Vizcaíno, 2013, 2015).

While these macroanatomical features have been well studied by mammalogists, the functional link between bone microstructure and fossoriality in mammals has received little attention in recent literature. Bone histology has long been known to be a powerful tool to investigate growth patterns and many other lifestyle parameters in vertebrates (see a review in de Ricqlès, 2011), and in recent years many studies have used bone microstructure as a proxy for inferring lifestyle in extant and extinct synapsids, both qualitatively (Ray, Botha & Chinsamy, 2004; Ray, Bandyopadhyay & Bhawal, 2009; Chinsamy & Hurum, 2006; Houssaye, 2009; Laurin, Canoville & Germain, 2011; Chinsamy-Turan, 2012; Amson et al., 2014) and quantitatively (Kriloff et al., 2008; Dumont et al., 2013; Quemeneur, de Buffrénil & Laurin, 2013; Laurin & de Buffrénil, 2016). Several studies have described the limb bone microanatomy and/or histology of extant burrowing mammals (Kolb et al., 2015). These studies, however, did not necessarily focus on adaptations linked with fossorial activity, instead describing bone microstructure in the context of growth patterns (Chinsamy & Hurum, 2006), sexual dimorphism and reproductive ability (Pinto et al., 2010), or evolutionary patterns (Straehl et al., 2013). More recently, a study on the long bone histology of the Cape dune molerat *Bathyergus suillus* (Montoya-Sanhueza & Chinsamy, 2017) described a high periosteal and endosteal cortical thickening through ontogeny for this species, and hypothesized it to be stimulated by its subterranean lifestyle. This represents the first time that fossorial behavior has been suggested as the main driving force behind a distinct histological feature, although other factors might also be involved in this increased cortical thickness (Montoya-Sanhueza & Chinsamy, 2017). These authors also identified in their specimens a notable amount of compacted coarse cancellous bone (CCCB), a bone tissue type formed through the compaction of trabeculae in the metaphysis, subsequently incorporated into the diaphyseal cortex during longitudinal growth (e.g. Enlow, 1963; see “Discussion”).

Over the last decade, a string of quantitative studies have focused on estimating bone compactness profiles of various long bones in tetrapods, using the specifically devoted software Bone Profiler (Girondot & Laurin, 2003), to build inference models able to identify differences in bone compactness and other related parameters (e.g., the *P* and *S* parameters; see Girondot & Laurin, 2003) for specific lifestyles, such as in aquatic/amphibious (Germain & Laurin, 2005; Kriloff et al., 2008; Canoville & Laurin, 2009, 2010; Quemeneur, de Buffrénil & Laurin, 2013; Houssaye et al., 2013, 2014a; Houssaye, Tafforeau & Herrel, 2014b; Houssaye, Fernandez & Billet, 2016a; Canoville & Chinsamy, 2015) or graviportal taxa (Houssaye, Fernandez & Billet, 2016a; Houssaye et al., 2016b). This approach, however, has only rarely been applied to fossorial taxa. Laurin, Canoville & Germain (2011) highlighted the difficulty in discriminating between fossorial and aquatic lifestyles in mammals from their bone microstructure, as opposed to the easier distinction between aquatic and terrestrial lifestyles. Accordingly, a large-scale study on the humerus of true moles (Meier et al., 2013) did not recover any pattern of bone compactness related to biomechanical constraints associated with fossoriality, despite the highly derived humeral morphology known to be linked with a subterranean lifestyle in that clade. However, two other parameters, the *S* value (i.e., the reciprocal of the slope of the compactness change between the cortical compacta and the spongiosa; Girondot & Laurin, 2003) and the ellipse ratio, presented a significant difference between fossorial and non-fossorial talpids, suggesting that a functional signal linked with fossoriality can be detected for some histological quantitative measurements. A large-scale study performed on a sample of extant and extinct xenarthrans (Straehl et al., 2013) also quantified bone compactness parameters, and identified a high difference between humeral and femoral compactness in some species, most notably in armadillos and anteaters. Whereas the authors hypothesized that this difference was linked with digging adaptations, they also acknowledged the need for larger fossorial taxa, such as the giant armadillo (*Priodontes maximus*) or mylodontid ground sloths, to be included in future studies to further investigate this hypothesis (although the hypothesis of fossorial behavior in mylodontids has yet to be confirmed; Vizcaíno et al., 2001).

Indeed, ecological constraints associated with a fossorial lifestyle, such as food availability or amount of soil to be excavated during burrowing, are known to be strongly dependent on body size and mass (Vizcaíno, Fariña & Mazzetta, 1999; Vizcaíno & Milne, 2002; Van Vuren & Ordeñana, 2012). A high allometric constraint on fossoriality has been documented in all burrowing mammals, both for the energetic cost of burrowing and for burrow size (McNab, 1979; Vleck, 1981; White, 2005; Noonan et al., 2015). For this reason, the fact that histological studies performed on digging mammals so far have focused almost exclusively on smaller species (body size <1 m; body mass <15 kg; an exception being the giant anteater, *Myrmecophaga tridactyla*, included in the sample of Straehl et al., 2013) might explain the low ecological signal identified in their bone microstructure, and larger mammals are likely to present a more significant signal associated with a greater burrowing cost.

The largest extant fossorial mammal is the aardvark, *Orycteropus afer* (Pallas, 1766), with a total body size ranging from 1.4 to 2.2 m, and a body mass from 40 to 100 kg (Shoshani, Goldman & Thewissen, 1988; Vizcaíno et al., 2001). The aardvark is the only extant representative of the order Tubulidentata, which falls under the large superorder Afrotheria, also comprising the elephants, sirenians, hyraxes, elephant shrews, golden moles, and tenrecs (Shoshani, Goldman & Thewissen, 1988; Svartman & Stanyon, 2012). It is endemic to sub-Saharan Africa, and has a strictly myrmecophagous diet (Melton, 1976; Taylor & Skinner, 2004). In order to survive as a large ant-eating endotherm living in a semi-arid environment with extreme variations in temperature, the aardvark has developed a series of unique ecological and physiological adaptations (Taylor & Skinner, 2004). The most significant of them is a highly specialized fossorial lifestyle that requires digging large burrows—on a daily basis for food or shelter, and less frequently for permanent residence—coupled with a nocturnal lifestyle that allows it to escape the high diurnal temperatures of its environment (Melton, 1976; Shoshani, Goldman & Thewissen, 1988; Taylor & Skinner, 2004). As a scratch-digger, the aardvark uses primarily its forelimbs to dig, with the hind limbs pushing back the resulting crushed soil, and the tail being used as the main support of the body weight (Endo et al., 2002, 2003, 2012). A typical burrow is about two–three m long and one m wide (Melton, 1976; Shoshani, Goldman & Thewissen, 1988), and can be as deep as six m under the soil surface, which makes it one of the largest burrows among mammals (White, 2005), and the deepest among all tetrapods (Platt et al., 2016). For this reason, the aardvark has been the subject of many comparative studies on its anatomy and physiology in the context of fossorial behavior, and the skeletal morphology and myology of its limbs in particular have been extensively studied (Humphry, 1868; Galton, 1869b; Sonntag, 1925; Sonntag & Woollard, 1925; Le Gros Clark & Sonntag, 1926; Thewissen & Badoux, 1986; Endo et al., 2002, 2003; Voegele, 2014). The inner structure of its limb bones, however, has never been described, which makes the aardvark a good candidate for a case study on the link between fossoriality and limb bone histology in mammals.

This study presents a histological description of the midshaft of all six limb bones of the aardvark in a functional context, with the aim of testing whether its lifestyle has an observable effect on its bone microstructure. Our goal is to describe any potential markers of fossoriality in the bone histology of this species. Histological features are discussed with respect to previous descriptions of the skeletal anatomy, myology, and ecology of the aardvark (Thewissen & Badoux, 1986; Endo et al., 2002, 2003; Voegele, 2014). Hypotheses on the relative and conjunct influences of biomechanical and other physiological signals on bone microstructure (Ruff, Holt & Trinkaus, 2006), and potential link between those signals and fossorial behavior, are also discussed.

## Materials and Methods

### Material

Despite a wide distribution across the continent of Africa, aardvark are rare components of ecosystems and given their nocturnal and secretive habits, they are difficult to collect, which has resulted in few specimens in museum collections (Skinner & Smithers, 1990). As thin sectioning is a destructive process and aardvark museum specimens are exceptionally rare, we were only permitted to section all six limb bones (i.e., humerus, radius, ulna, femur, tibia, and fibula) from the right side of three specimens, with the addition of the three bones from the left forelimb of one specimen (i.e., humerus, radius, and ulna; see Table 1), the left hind limb of which was missing. The provision of these specimens has provided the first opportunity to study the bone histology of this species. All specimens were originally captured in the wild. Collection registers allowed the identification of two specimens as being adults, and the third one as being a subadult (there are currently only two juvenile specimens in South African museum collections, neither of which we were permitted to thin section). No information relative to the body mass, size, sex, or precise location of collection for the specimens was available; we performed several osteoanatomical measurements to ensure all specimens belong to a similar size range. The specimen labeled as a subadult (NMBF 12311) presents values slightly lower than that of the other two for these measurements, which might indicate that it died at a younger age (see Table 1). However, all specimens present completely fused epiphyses for all bones, which can be interpreted as evidence for them to be fully grown, or nearly fully grown, individuals (Karaplis, 2008; Martin et al., 2015). Complete cast replicas of each sampled bone were produced prior to histological sectioning, using standard molding and casting techniques (Lamm, 2013). Additionally, photographs of each bone in anterior and posterior view (or lateral and medial view for the ulna, so that the olecranon and coronoid processes appear more clearly) were taken using a Canon EOS 760D digital camera (Tokyo, Japan).

**Table 1:** Collection and anatomical information for sampled specimens.

Specimen	Ontogenetic stage	Sampled bones	Locality of collection	Right humerus length (mm)	Right humerus midshaft mediolateral width (mm)	Right femur length (mm)	Right femur midshaft mediolateral width (mm)
MVD-M 1	Adult	Left and right forelimbs, right hind limb	Northern Cape	145.36	15.84	192.84	29.87
MMK 7243	Adult	Right forelimb and hind limb	Northern Cape	146.11	18.63	190.81	20.79
NMBF 12311	Subadult	Right forelimb and hind limb	Free State	143.97	15.67	189.71	20.21

**Note:**

Sampled bones include the humerus, radius, and ulna (forelimb), and femur, tibia, and fibula (hind limb).

### Methods

**Figure 1:**
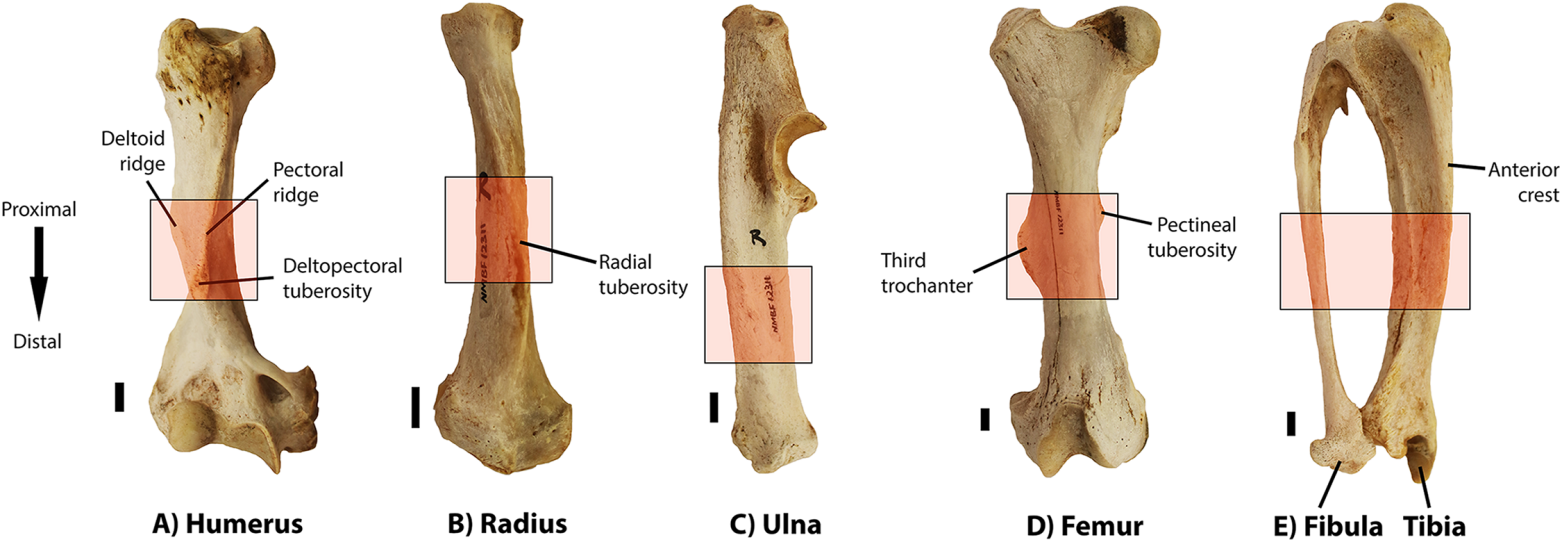
Photographs of all six limb bones from the right side of the specimen NMBF 12311, showing the zones (red rectangles) in the midshaft that were sampled for serial cross-sectioning for each of them. Tibia and fibula are pictured together, as these two bones are proximally fused in the aardvark. All bones are presented in anterior view, except the ulna, which is shown in lateral view due to its mediolaterally flattened shape (see text). Macroanatomical structures mentioned in the histological descriptions (Results section) are labelled. (A) Humerus. (B) Radius. (C) Ulna. (D) Femur. (E) Tibia and fibula. Scale bar: one cm.

All 21 sampled bones were cut transversally into smaller portions of one–two cm long using a Makita^®^ CC300DW cordless cutter, and degreased using liquid soap to remove fatty tissue. After a period of drying, they were then processed through a week-long pretreatment procedure, using a Sakura Tissue-Tek^®^ (Torrance, CA, USA) VIP E150–300 vacuum infiltration processor. The pretreatment sequence includes fixation in 10% buffered formalin (48 h), 70% ethanol (48 h), 90% ethanol (48 h) to remove excess water, and xylene (24 h), here used as a clearing agent. The bones were then embedded under vacuum in Struers EpoFix^®^ resin. The resulting blocks were left to dry for 48 h, serially sectioned into approximately 1.5 mm thick cross-sections using a Struers Accutom-100^®^, and adhered to two or five mm-thick plastic slides (depending on the size of the bone) with EpoFix^®^ resin (Cleveland, OH, USA). In order to consider intraspecimen variability between the proximal and distal parts of the midshaft (Lamm, 2013), we serially cross-sectioned the whole diaphysis of all the bones (Fig. 1). Sections were performed at about 40–60% from the proximal articular surface for the humerus, tibia, and fibula, and at about 35–55% for the radius, ulna, and femur. Around 7–10 sections were obtained for each of the 21 sampled midshafts, and more than 180 histological sections were thus prepared for this study.

Thick sections were then ground further using the Struers Accutom-100^®^, to a thickness of 70–100 μm. Resulting thin sections were then digitally rendered under ordinary, polarized, and cross-polarized (CPL) light, using polarizing microscopes (Nikon Eclipse 50i POL, Nikon Eclipse Ci-POL) equipped with digital cameras (DS-Fi1 and DS-Fi3, respectively), in NIS-Elements 4.5 (Nikon Corp., Tokyo, Japan). Composite images of complete slide scans were automatically assembled in NIS-Elements, and figures using these images and close-ups showing details of histological structures (see “Results”) were compiled in Photoshop CS6 (Adobe Systems Inc., San Jose, CA, USA). Bone histological terminology and definitions generally follow that of Enlow (1963), Francillon-Vieillot et al. (1990), de Ricqlès et al. (1991) and Chinsamy-Turan (2005).

## Results

**Figure 2:**
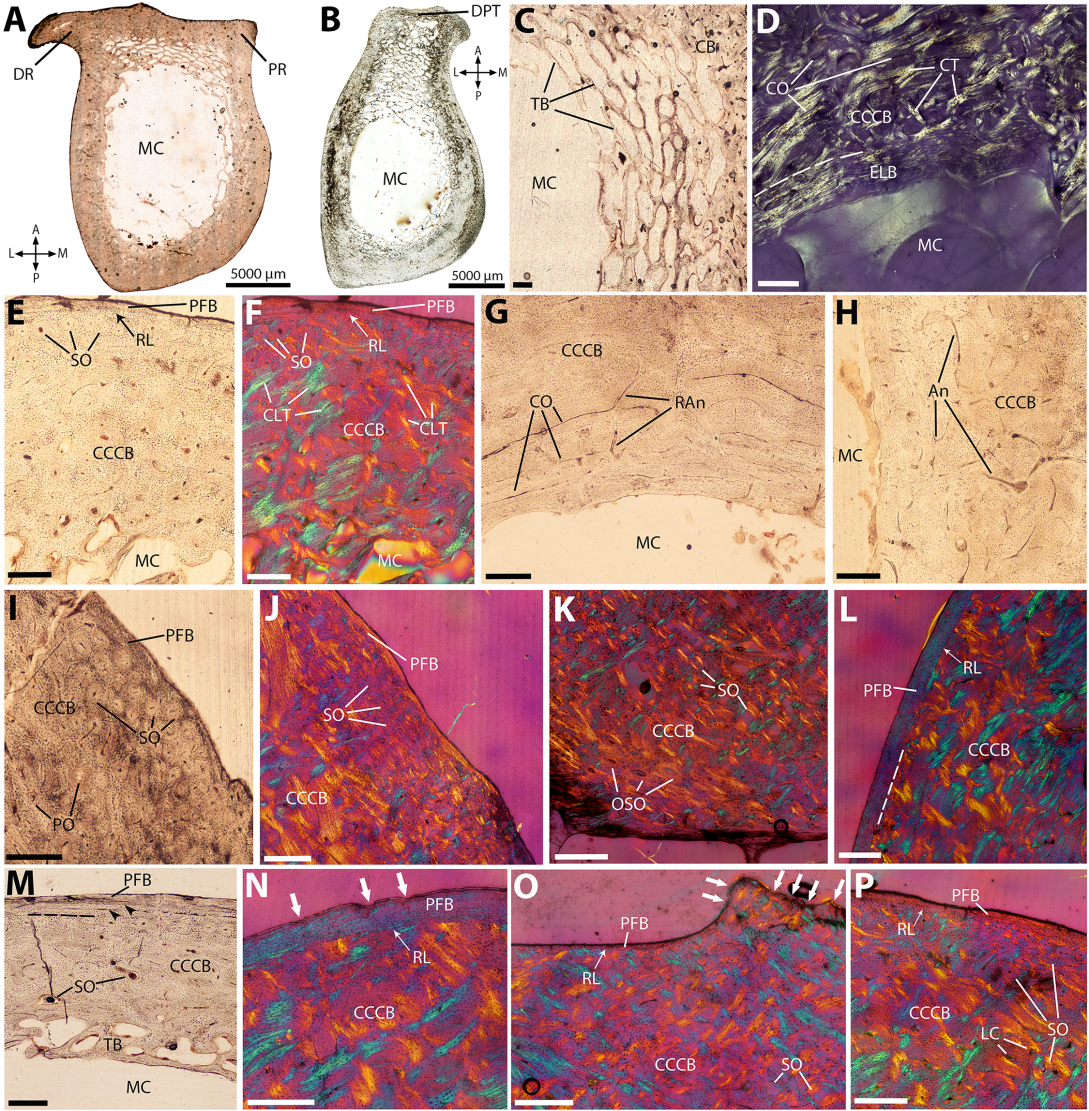
Histological cross-sections of humeri, under OL (A–C, E, G–I, M), PL (D), and CPL (F, J–L, N–P). (A) Whole cross-section of the proximal midshaft, showing the large deltoid (DR) and pectoral (PR) ridges. (B) Whole cross-section of the distal midshaft, showing the elongated deltopectoral tuberosity (DPT). (C) Detail of the trabecular network on the anterior side. (D) Endosteal lamellar bone in the innermost cortex, on the lateral side. (E) Cortical bone showing extensive compacted coarse cancellous bone (CCCB), with a thin outer layer of parallel-fibered bone (PFB), and a resorption line between the two. (F) Same as previous image, but under CPL, showing the characteristic mesh-like structure of compacted trabeculae in CCCB, appearing as blue and yellow struts perpendicular to each other. (G) Detail of the vascular pattern in the CCCB of the inner cortex, showing mostly circular canals and radial anastomoses. (H) Same as previous image, but with a more reticular pattern due to more anastomoses. (I) Detail of the densely packed secondary osteons in the outer cortex. (J) Same as previous image, but under CPL, showing the CCCB still visible in-between secondary osteons. (K) Detail of the local pattern of oblique secondary osteons in the outer cortex of the deltopectoral tuberosity. (L) Outer PFB layer, separated from CCCB by a resorption line. (M) Two lines of arrested growth (LAGs), indicated by black arrowheads, in the outer cortex; in most sections, only one LAG is visible (see text). (N) Detail of the resorption line between CCCB and PFB, and thick PFB layer with scalloped outer edge (white arrows), indicating periosteal resorption, on the lateral surface. (O) Detail of the very thin, almost entirely resorbed PFB layer on the medial edge, with strong periosteal resorption (white arrows). (P) Very thin PFB layer on the posterior side. A, P, L, M (in A and B): anterior, posterior, lateral, and medial orientations; An, anastomoses; CB, cortical bone; CCCB, compacted coarse cancellous bone; CLT, compacted lamellar trabeculae; CO, circumferentially-oriented osteons; CT, compacted trabeculae; DPT, deltopectoral tuberosity; DR, deltoid ridge; ELB, endosteal lamellar bone; LC, longitudinal canals; MC, medullary cavity; OSO, obliquely-oriented secondary osteons; PFB, parallel-fibered bone; PO, primary osteons; PR, pectoral ridge; RAn, radial anastomoses; RL, resorption line; SO, secondary osteons; TB, trabecular bone. Dashed lines indicate resorption lines. Scale bar for C–P: 0.5 mm.

**Figure 3:**
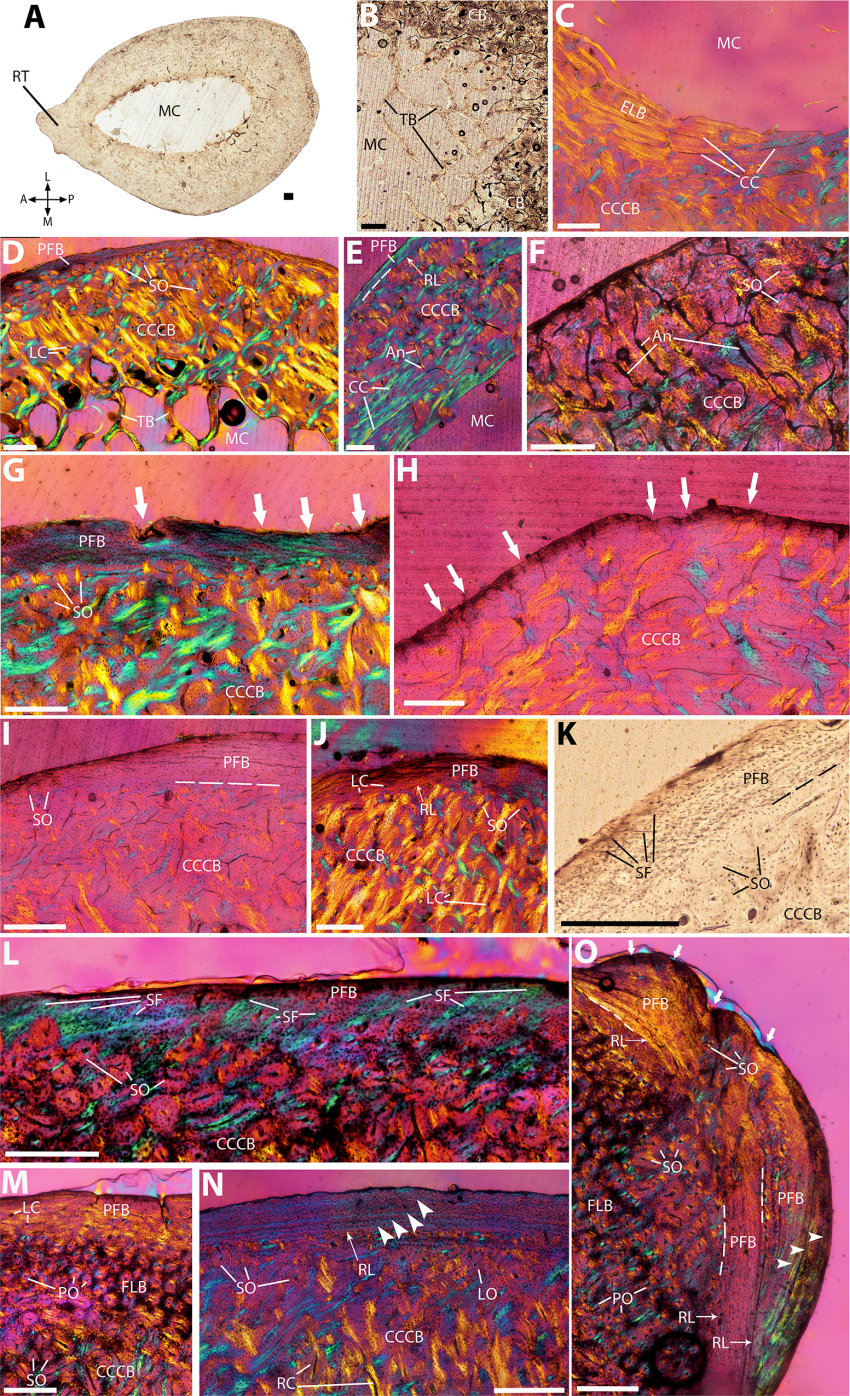
Histological cross-sections of radii, under OL (A–B, K) and CPL (C–J, L–O). (A) Whole cross-section of the mid-diaphysis, with the radial tuberosity protruding on the anteriorside. (B) Detail of the trabeculae on the posterior side. (C) Endosteal lamellar bone in the innermost anteromedial cortex. (D) Whole medial transect, showing the predominance of CCCB across the cortex. (E) Detail of the circular canals and radial anastomoses in the inner cortex. (F) Detail of the dense network of anastomoses in the outer part of cortical CCCB. (G) Secondary osteons forming dense patches at the junction between CCCB and PFB, entirely covering the resorption line between the two; white arrows indicate local periosteal resorption. (H) Detail of the periosteal resorption front (white arrows), and subsequent lack of PFB outer layer, on the anterior side. (I) Detail of the PFB layer on the lateralside, showing an increase in thickness from the anterolateral (left) to the posterolateral (right) side. (J) Detail of the posterior side, where the PFB layer is the thickest. (K) Detail of the Sharpey’s fibers in the PFB outer layer of the posteromedial side, in NMBF 12311. (L) Large bundles of Sharpey’s fibers in the PFB outer layer of the posteromedial orientation, in MVD-M 1. (M) Patch of FLB with longitudinal osteons, between the CCCB and PFB on the posterolateral side. (N) Detail of the outer cortex, showing a periosteal resorption line separating CCCB from PFB, as well as three LAGs (white arrowheads) in the PFB. (O) Outer bulge of PFB in MVD-M 1 showing three conspicuous LAGs (white arrowheads) and periosteal resorption (white arrows on the periosteal edge), on top of the PFB layer on the posteromedial side; the two PFB layers are separated from the underlying patch of FLB by a resorption line, and from each other by another one. A, P, L, M (in A): anterior, posterior, lateral, and medial orientations; An, anastomoses; CB, cortical bone; CC, circular canals; CCCB, compacted coarse cancellous bone; ELB, endosteal lamellar bone; FLB, fibrolamellar bone; LC, longitudinal canals; LO, longitudinally-oriented osteons; MC, medullary cavity; PFB, parallel-fibered bone; PO, primary osteons; RC, radial canals; RL, resorption line; RT, radial tuberosity; SF, Sharpey’s fibers; SO, secondary osteons; TB, trabecular bone. Dashed lines indicate resorption lines. Scale bar: 0.5 mm.

**Figure 4:**
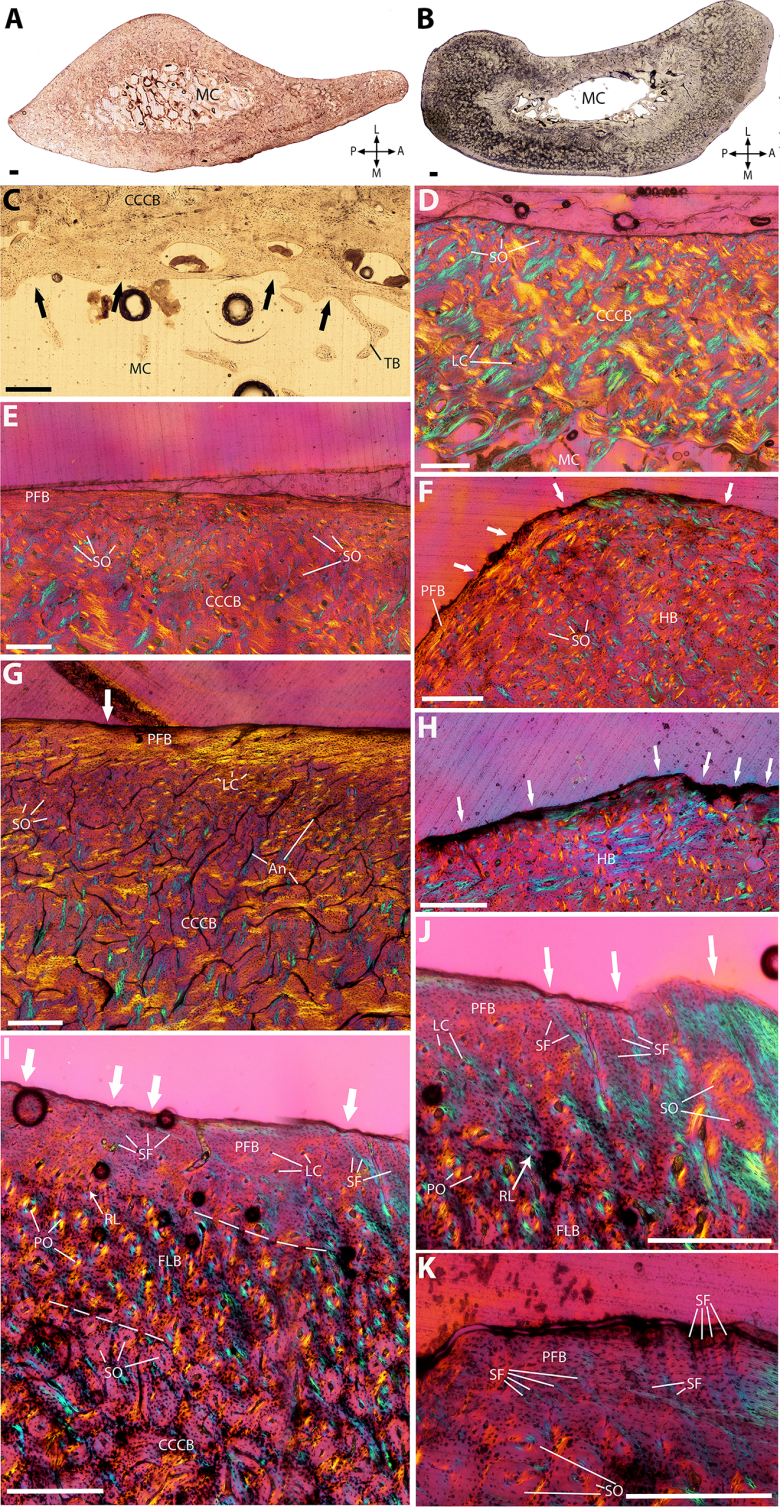
Histological cross-sections of ulnae, under OL (A–C) and CPL (D–K). (A) Whole cross-section of the proximal midshaft, showing the anterolateral concave periosteal edge. (B) Whole cross-section of the distal midshaft, showing a thicker anterior and posterior cortex than the proximal one. (C) Innermost cortex, showing signs of endosteal resorption (black arrows). (D) Cortex of the lateral side, consisting entirely of CCCB with many secondary osteons. (E) Detail of Haversian remodeling at the limit between CCCB and PFB, in the anteromedial orientation. (F) Dense Haversian bone (HB) in the outer cortex of the posterior orientation, showing high periosteal resorption (white arrows). (G) Detail of the limit between CCCB and PFB on the posterolateral side, showing many secondary osteons, and local periosteal resorption (white arrow). (H) Detail of the scalloped periosteal edge of the posterior surface, due to periosteal resorption (white arrows). (I) Patch of FLB sandwiched in-between CCCB and FLB, on the anterior side; the PFB layer shows strong periosteal resorption (white arrows), and presents several Sharpey’s fibers. (J) and (K) Detail of the radially-oriented Sharpey’s fibers, in the PFB layer on the anteromedial side, also showing periosteal resorption (white arrows). A, P, L, M (in A and B): anterior, posterior, lateral, and medial orientations; An, anastomoses; CCCB, compacted coarse cancellous bone; FLB, fibrolamellar bone; HB, Haversian bone; LC, longitudinal canals; LSO, longitudinally-oriented secondary osteons; MC, medullary cavity; PFB, parallel-fibered bone; PO, primary osteons; RL, resorption line; SF, Sharpey’s fibers; SO, secondary osteons; TB, trabecular bone. Dashed lines indicate resorption lines. Scale bar: 0.5 mm.

**Figure 5:**
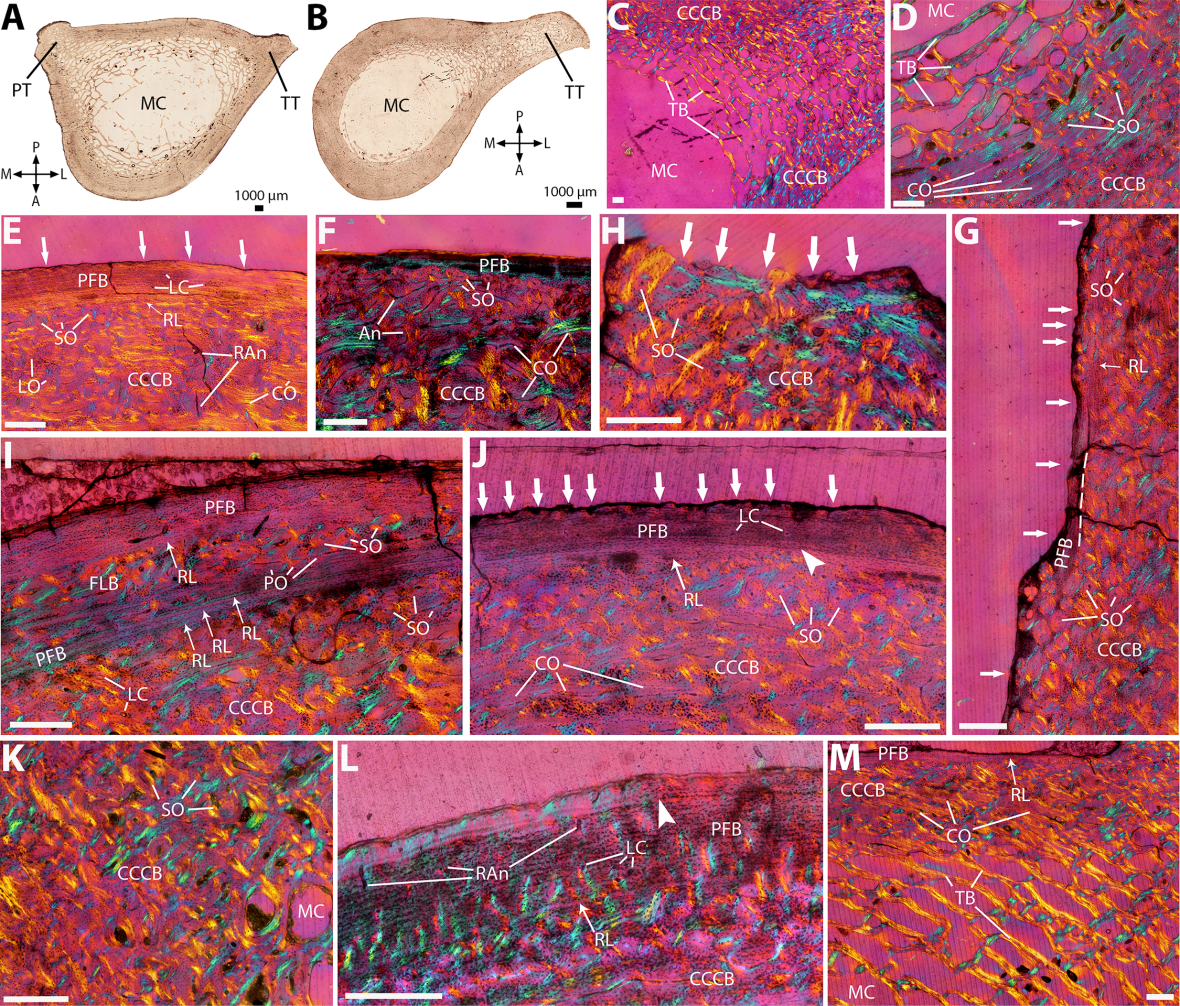
Histological cross-sections of femora, under OL (A–B) and CPL (C–M). (A) Whole cross-section of the proximal midshaft, showing acute angles of the posterior ridge with the adjacent lateral and medial sides. (B) Whole cross-section of the distal midshaft, showing the enlarged third trochanter protruding laterally. (C) Detail of the dense trabecular network in the third trochanter. (D) Large circular osteons in the inner cortex. (E) Mid-cortical CCCB showing a variety of vascular canals (longitudinally- and circumferentially-oriented osteons, radial anastomoses, and secondary osteons), and outer layer of PFB with periosteal resorption (white arrows). (F) Same as (E), with a higher proportion of anastomoses forming a more reticular vascular pattern. (G) High periosteal resorption (white arrows) of CCCB, on the medial side. (H) Strong periosteal resorption (white arrows) on the lateral edge of the third trochanter. (I) Thin layer of highly remodeled FLB positioned in the outer PFB layer on the posterior side. Three resorption lines indicate that these outer layers of FLB and PFB were formed after the endosteal deposition of CCCB. Many secondary osteons are visible in the CCCB and FLB, and a resorption line separates FLB from outermost PFB. (J) Thick outer layer of PFB on the anterior side, showing strong periosteal resorption (white arrows) and a LAG (white arrowhead). (K) Highly compact and remodeled CCCB in the posteromedial cortex. (L) Detail of the longitudinal canals and radial anastomoses in the outer PFB layer on the anterior side, showing one LAG (white arrowhead). (M) Elongated, circumferentially-oriented osteons in the inner cortex of the third trochanter, matching the orientation of the underlying trabeculae in the medulla. A, P, L, M (in A and B): anterior, posterior, lateral, and medial orientations; An, anastomoses; CCCB, compacted coarse cancellous bone; CO, circumferentially-oriented osteons; FLB, fibrolamellar bone; LC, longitudinal canals; LO, longitudinally-oriented osteons; MC, medullary cavity; PFB, parallel-fibered bone; PO, primary osteons; PT, pectineal tuberosity; RAn, radial anastomoses; RL, resorption line; SO, secondary osteons; TB, trabecular bone; TT, third trochanter. Dashed lines indicate resorption lines. Scale bar for C–M: 0.5 mm.

**Figure 6:**
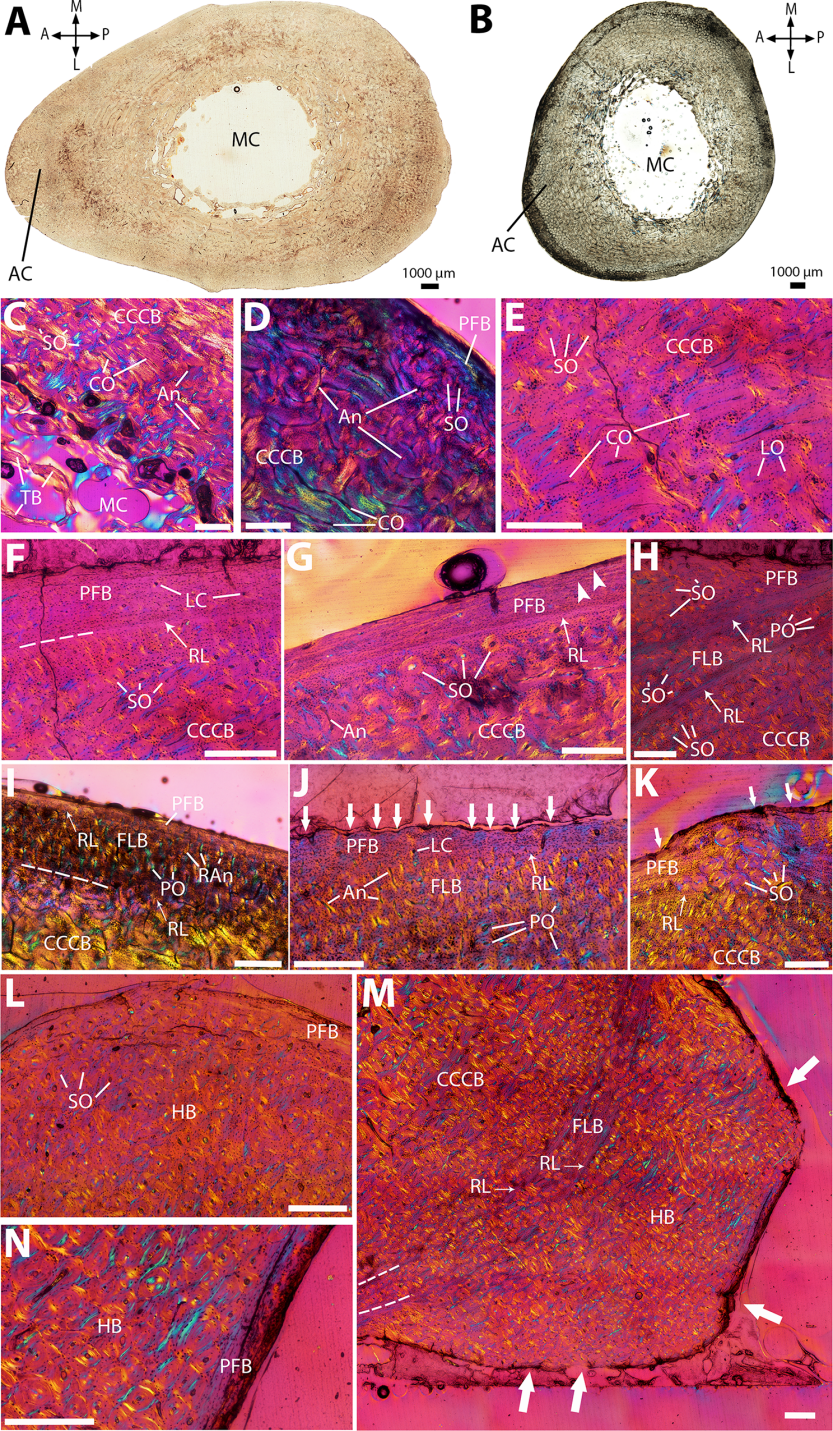
Histological cross-sections of tibiae, under OL (A–B) and CPL (C–N). (A) Whole cross-section of the proximal midshaft, showing the enlarged anterior crest. (B) Whole cross-section of the distal midshaft, showing a more reduced anterior crest and an overall more circular section shape. (C) CCCB with circumferentially-oriented osteons and secondary osteons, in the inner cortex. (D) Reticular vascular pattern in the CCCB of the mid-cortex. (E) Detail of Haversian remodeling in the mid-cortex. (F) PFB layer in the outer cortex, separated from CCCB by a resorption line. (G) Thin outer layer of PFB with two LAGs (white arrowheads), on the medial side. (H) Thin layer of FLB wedged between CCCB and PFB, all three of them being highly remodeled—FLB and PFB could almost be described as HB. A resorption line separates FLB from CCCB, and another one lies between FLB and PFB. (I) Radial vascular pattern in the FLB layer, due to many radial anastomoses, on the posterior side. (J) Highly scalloped PFB (white arrows), indicating strong periosteal resorption. (K) Local pervading of the outer cortex by densely packed longitudinal secondary osteons, on the posteromedial side; PFB is extensively eroded on its periosteal edge (white arrows). (L) HB in the outer cortex on the anterior side, where the anterior crest is the most developed. (M) Outer bulge of highly resorbed (white arrows) HB on the outer edge of the posterior side, in NMBF 12311. A resorption line lies between CCCB and a thin layer of remodeled FLB, and another one separates this FLB layer from the outer bulge of HB. (N) Detail of (M) showing an outer patch of unremodeled PFB in the HB bulge. A, P, L, M (in A and B): anterior, posterior, lateral, and medial orientations; AC, anterior crest; An, anastomoses; CCCB, compacted coarse cancellous bone; CO, circumferentially-oriented osteons; FLB, fibrolamellar bone; HB, Haversian bone; LC, longitudinal canals; LO, longitudinally-oriented osteons; MC, medullary cavity; PFB, parallel-fibered bone; PO, primary osteons; RAn, radial anastomoses; RL, resorption line; SO, secondary osteons; TB, trabecular bone. Dashed lines indicate resorption lines. Scale bar for C–N: 0.5 mm.

**Figure 7:**
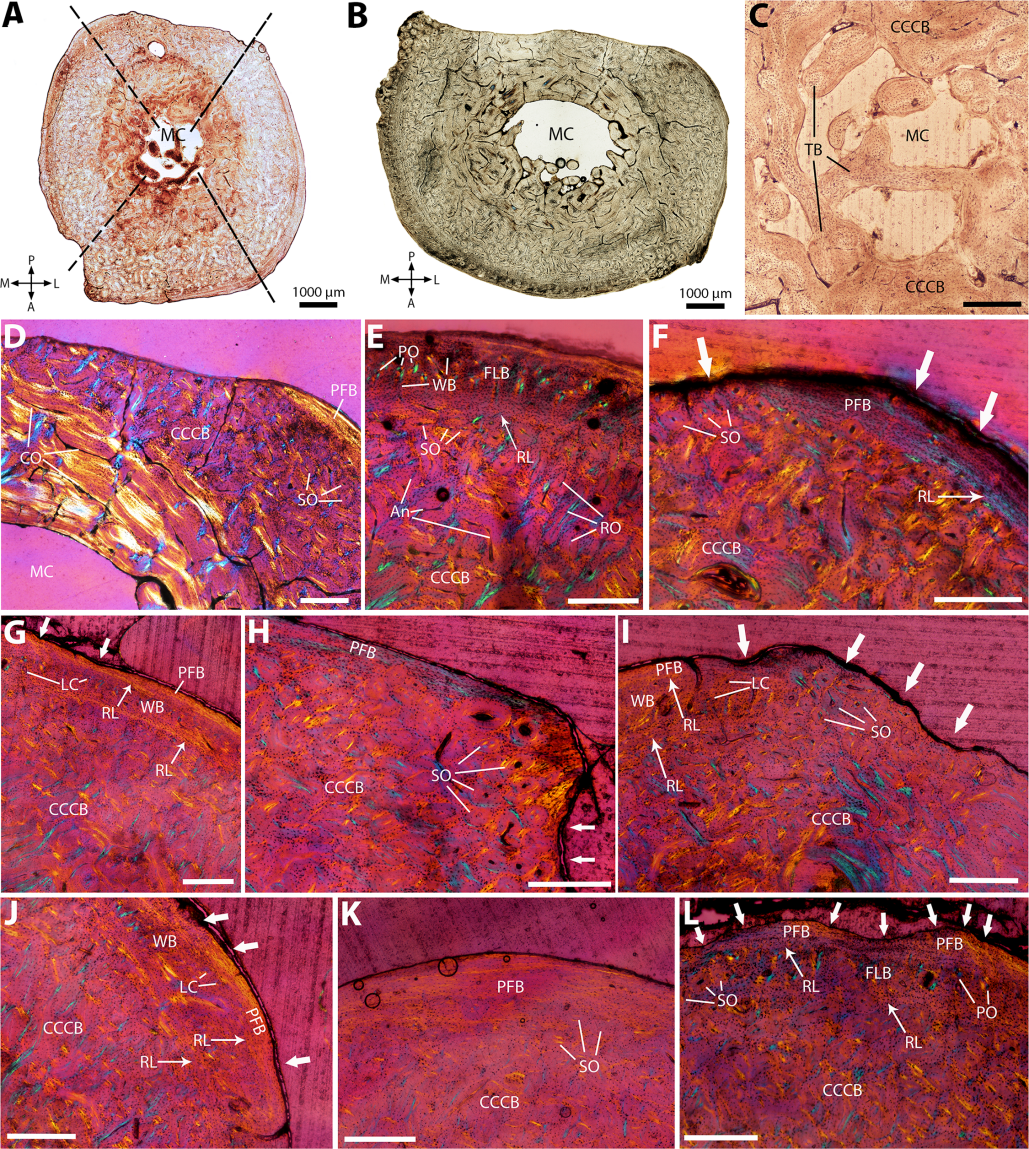
Histological cross-sections of fibulae, under OL (A–C) and CPL (D–L). (A) Whole cross-section of the proximal midshaft, showing an overall circular section shape. (B) Whole cross-section of the distal midshaft, showing a strong periosteal resorption on the posterior side. Dashed lines show the limits between the four main sides of the section, as its roughly circular outline does not allow to visualize them as easily as in other limb bones in the sample. (C) Detail of the small medullary cavity, with a few disorganized trabeculae. (D) CCCB with circumferentially-oriented osteons and a few secondary osteons, on the posterior side; only a thin outer layer of PFB is visible in the outer cortex. (E) CCCB in the mid-cortex, showing a variety of vascular canals (radially-oriented osteons, many oblique anastomoses, and secondary osteons on its outer limit). A layer of FLB lies on top of it, and is itself topped by a layer of PFB, all layers being separated from each other by resorption lines. (F) Detail of the many secondary osteons at the outer limit of CCCB, and of the scalloped outline (white arrows) of the outer PFB layer. (G) WB layer topping CCCB with a resorption line in-between, and thin, extensively resorbed (white arrows) outer PFB layer on top of it. (H) and (I) Detail of the patches of densely packed secondary osteons locally protruding at the limit between main orientations, on the posteromedial side for (H), and on the posterolateral side for (I). White arrows indicate periosteal resorption. (J) Same as (I), but on the medial side, showing a thicker outer layer of PFB. (K) Detail of the PFB layer on the anterolateral side, where it is the thickest. (L) Same as (K), but on the posteromedial side, where PFB is almost entirely resorbed (white arrows), and a thick layer of FLB is visible on top of CCCB, separated from it by a resorption line. A, P, L, M (in A and B): anterior, posterior, lateral, and medial orientations; An, anastomoses; CCCB, compacted coarse cancellous bone; CO, circumferentially-oriented osteons; FLB, fibrolamellar bone; LC, longitudinal canals; MC, medullary cavity; PFB, parallel-fibered bone; PO, primary osteons; RL, resorption line; RO, radially-oriented osteons; SO, secondary osteons; TB, trabecular bone; WB, woven bone. Scale bar for C–L: 0.5 mm.

All descriptions were obtained from histological thin sections prepared and hosted at the Karoo Palaeontology Department in the National Museum, Bloemfontein, as depicted in Figs. 2–7. We sampled homologous bones from both the left and right side for one of our specimens (MVD-M 1); since left and right elements present similar histological characteristics, we do not consider sidedness to have a significant influence on the histological profile of the bones in our sample (see Woodward, Horner & Farlow, 2014). Similarly, the histological profile of one particular bone did not show much variation between sections taken from different parts of its midshaft. Hence the general description for each of the six limb bones included in this study applies to all corresponding specimens in the sample, unless mentioned otherwise.

### Humerus

Cross-sections present an ovoid shape, with two well-developed ridges on the anterior side, due to the strong protrusion of the deltoid and pectoral ridges at the diaphyseal level (Le Gros Clark & Sonntag, 1926; see also Lehmann et al., 2006). In the proximalmost sections, those ridges are distinct from each other (Fig. 2A), whereas in the more distal part of the midshaft, they are fused in a large deltopectoral tuberosity, resulting in an overall ‘bottleneck-like’ section shape (Fig. 2B). The cortex is relatively thin, and of even thickness throughout the section (Figs. 2A and 2B). The medullary cavity is large and well-defined, with a dense network of bony trabeculae packing its anterior side in the distal midshaft, underlying the protruding deltopectoral tuberosity (Figs. 2B and 2C). Some trabeculae are also present on its medial side (Figs. 2A and 2B). The innermost part of the cortex presents endosteal lamellar bone, mostly visible on the lateral side (Fig. 2D). The inner and mid-cortex consists of CCCB, which is formed through the compaction of trabeculae in the metaphysis, subsequently incorporated into the diaphyseal cortex during longitudinal growth (Fig. 2E; see Discussion). CCCB presents a characteristic structure of compacted lamellar trabeculae, oriented either in parallel or perpendicularly to the periosteum, resulting in a mesh-like structure that is most conspicuous when observed under CPL (Fig. 2F). Most vascular canals in the inner cortex are circumferentially oriented, with a few radial anastomoses (Fig. 2G), and can locally form a more reticular pattern (Fig. 2H).

Vascularization becomes mostly longitudinal in the mid-cortex, where Haversian remodeling becomes more prevalent (Fig. 2I). The pattern of longitudinal secondary osteons becomes denser closer to the periosteum, and the outer cortex is heavily remodeled (Fig. 2J). The underlying pattern of CCCB, however, is still visible in-between secondary osteons (Fig. 2J), and thus cannot be described as dense Haversian bone (HB). Secondary osteons are more numerous on the anterior and posterior side; in the deltopectoral tuberosity, some of them form a dense, obliquely-oriented pattern (Fig. 2K). The outmost cortex presents a thin layer of poorly vascularized parallel-fibered bone (PFB) on its lateral side (Fig. 2L), which contains at least one line of arrested growth (LAG), indicating a temporary cessation in growth (Fig. 2M). The periosteal edge of the PFB presents a clear resorption front, as does the outer edge of CCCB underneath this PFB layer (Fig. 2N). This contrasts with the outermost cortex on the medial side, where the PFB layer is almost completely resorbed, and the periosteal edge is more scalloped (Fig. 2O). On the anterior and posterior sides, the PFB layer, when present, is also very thin (Fig. 2P).

### Radius

The shape of the cross-sections is uneven, with the small radial tuberosity protruding on the anterior side (Fig. 3A), and a concave periosteal edge on the anterolateral side, where the cortex is thinner than in the rest of the section (Fig. 3A). The radial tuberosity protrudes more toward the distal end of the midshaft, otherwise, the general shape and histology of the sections do not show any conspicuous variation on the proximodistal axis of the bone. The medullary cavity is relatively small, of elliptic shape, and exhibits a loose trabecular network in the mid-diaphysis, mostly concentrated on the posterior side of the section (Fig. 3B). Endosteal lamellae are present in the innermost part of the cortex, and are thicker on the anterolateral and anteromedial sides (Fig. 3C). Most of the cortex is comprised of CCCB (Fig. 3D); vascular canals are mostly circular in the inner cortex (Fig. 3E), and from the mid-cortex form a much more reticular pattern, with many oblique anastomoses, as the compacted trabeculae become more densely packed (Figs. 3E and 3F). The outer edge of the CCCB layer is highly remodeled (Fig. 3G). The outer cortex presents a layer of poorly vascularized PFB, of uneven thickness through the section: absent on the anterior side, where the periosteal edge of the anterior tuberosity is highly scalloped (Fig. 3H), it thickens gradually toward the medial and lateral edges (Fig. 3I), to reach its maximum thickness on the posterior side (Fig. 3J). On the posteromedial side, the PFB layer presents Sharpey’s fibers (indicating areas of muscle insertion) in almost all sections, ranging from just a few in NMBF 12311 (Fig. 3K) to large bundles of them in MVD-M 1 (Fig. 3L), covering most of the outer cortex on this side. On the posterolateral side, the PFB layer is very thick, and there is a small patch of fibrolamellar bone (FLB) in-between the CCCB and PFB, containing many small longitudinal primary osteons (Fig. 3M). Three to four conspicuous LAGs can be observed in the PFB outer layer (Fig. 3N), on at least one side. In MVD-M 1, a small bulge of PFB lies on top of this PFB outer layer on the posterolateral side, separated from it by an additional resorption line (Fig. 3O), indicating strong periosteal resorption.

### Ulna

The sections are elongated on their anteroposterior axis, with a flattening of the medial and lateral sides of the cortex. The general shape is highly asymetrical, with a strong outward curvature on the lateral side, where the cortex is thinner than in the rest of the section (Fig. 4A). This curvature becomes more pronounced from the proximal to the distal part of the midshaft, along with a thickening of the cortex, especially in its anterior part (Fig. 4B). The spongiosa is well developed, especially in the proximalmost part of the midshaft (Figs. 4A and 4B). The medullary cavity is small, with its endosteal edges showing signs of previous resorption (Fig. 4C). The cortex consists almost entirely of CCCB with small longitudinal canals (Fig. 4D). Much of it is highly remodeled with longitudinal secondary osteons, the distribution of which becomes denser when closer to the periosteal surface (Fig. 4E); this pattern is especially present on the posterior and anterior sides, where the periosteal edges of CCCB present patches of dense HB (Fig. 4F). A thin layer of PFB tops the outermost cortex; in the proximal midshaft, it is only present in the posterolateral and anteromedial sides (Fig. 4G), whereas the rest of the outer cortex presents a scalloped outer edge, indicating periosteal resorption (Fig. 4H). Conversely, in the distal midshaft, the PFB layer becomes thicker on the anterior side, and is slightly more vascularized with small longitudinal canals (Fig. 4I). A small patch of FLB can be seen between the CCCB and PFB on the anterior side (Fig. 4I), whereas the PFB on the posterior side has been completely eroded through periosteal resorption (Fig. 4H). The anteromedial part of the PFB layer presents many radially-oriented Sharpey’s fibers (Figs. 4I, 4J and 4K). Growth marks are absent.

### Femur

The sections are irregular in shape, with a predominant circular outline and a flattened ridge on the posterior side (Fig. 5A). In the proximal part of the midshaft, this ridge forms two acute angles with the adjacent medial and lateral sides (Fig. 5A). The medial angle corresponds to the pectineal tuberosity, that is, the attachment site for the pectineus muscle (Sonntag, 1925); toward the mid-diaphysis, this tuberosity progressively disappears, whereas the lateral angle protrudes even more as the third trochanter becomes more prominent (Fig. 5B; Le Gros Clark & Sonntag, 1926). The medullary cavity is large, and cortical thickness remains relatively constant through the whole section (Figs. 5A and 5B). A dense network of trabeculae can be observed under each angle formed by the posterior ridge (Fig. 5A), as well as consolidating the extension of the third trochanter in more distal sections (Figs. 5B, 5C and 5M). The inner and mid-cortex consists of CCCB, with circumferentially-oriented lamellae and vascular canals in the inner cortex (Fig. 5D), and a denser, more irregular vascularization in the mid-cortex, where secondary remodeling becomes more prominent (Fig. 5E). Locally, this vascularization may form a reticular pattern (Fig. 5F).

In the outer cortex, the CCCB becomes almost completely remodeled, and its periosteal edge shows resorption on the lateral and medial sides (Fig. 5G), particularly on the third trochanter where it is highly scalloped (Fig. 5H), whereas it is topped by a layer of PFB in the posterior and anterior sides. In all sections, there is a thin, highly remodeled FLB layer sandwiched inside the PFB one, on the posterior side (Fig. 5I). The very thin PFB layer between the FLB and CCCB presents three resorption lines (Fig. 5I), suggesting that the periosteal growth of the FLB layer took place after the endosteal formation of CCCB (Enlow, 1963; de Ricqlès et al., 1991). A fourth resorption line separates the FLB from the outer PFB, which also presents signs of periosteal resorption, suggesting a succession of multiple resorption events, which took place after the deposit of the outer PFB on top of the FLB (Fig. 5I). This resorption is likely to have entirely eroded the FLB layer in the rest of the section. On the anterior side, the PFB layer is thick, but scalloped; it contains a few small longitudinal canals, and at least one LAG is visible (Fig. 5J). The angles formed by the posterior ridge in the proximal midshaft present CCCB with a very dense and irregular vascular pattern (Figs. 5H and 5K); the outer PFB layer is almost completely resorbed (Fig. 5H), and many longitudinal secondary osteons can be observed (Fig. 5K). The PFB in general only has a few longitudinal canals and radial anastomoses (Fig. 5L). In the lateral extension of the third trochanter, the CCCB presents a pattern of very elongated, circumferentially-oriented osteons (Fig. 5M), matching the side of the trabecular network in the underlying spongiosa (Figs. 5M and 5C).

### Tibia

The proximalmost sections have an ovoid shape, with a prominent anterior extension corresponding to the anterior crest, running from the proximal epiphysis to the mid-diaphysis (Le Gros Clark & Sonntag, 1926; Fig. 6A). The more distal sections present a more rounded shape, although the cortex is still slightly thicker on the anterior side than in the rest of the section (Fig. 6B). The medullary cavity is reduced and flattened on its anteroposterior axis, with only a few disorganized trabeculae (Figs. 6A and 6B). The inner cortex consists of CCCB with a mostly circular vascular pattern and a few secondary osteons (Fig. 6C). In the mid-cortex, vascularization becomes more reticular, with many radial and oblique anastomoses (Fig. 6D), along with more prominent Haversian remodeling (Fig. 6E). Many secondary osteons are aligned along the limit between the CCCB and the outer cortex (Fig. 6F), marked by a thin layer of PFB with at least one resorption line (Fig. 6F).

The outer cortex, as in the femur, consists of two distinct layers. On the lateral and medial sides, only a relatively thin layer of PFB, with at least two LAGs, is present—likely because of periosteal resorption, since the CCCB layer ends externally with a resorption line (Fig. 6G). On the anterior and posterior sides, however, the two layers are preserved. The inner one consists of mostly remodeled FLB with mainly small longitudinal osteons (Fig. 6H); on the posterior side, where this layer reaches its maximum thickness, many radial anastomoses can be observed in some sections (Fig. 6I). The outermost layer is almost avascular PFB (Fig. 6J), with periosteal edges showing signs of erosion on the whole section (Fig. 6J). Dense patches of secondary osteons can locally pervade the outer cortex up to its periosteal edges (Fig. 6K), particularly in the anterior crest, which is almost entirely remodeled in the proximalmost sections where it is most prominent (Fig. 6L). In NMBF 12311, an outer bulge of bone is present on top of the outer layer of PFB on the posterolateral side, separated from it by another resorption line (Fig. 6M), and consists almost entirely of dense HB (Fig. 6N). The few visible patches of primary bone consist of PFB (Fig. 6N), suggesting that the original PFB layer was much thicker than in the rest of the section, and has been almost entirely resorbed except for that bulge.

### Fibula

Sections present a roughly circular outline, with a flattened edge on the posterior side, and do not vary much in shape through the midshaft compared to other bones in the sample (Figs. 7A and 7B). The medullary cavity is very small, with a diameter inferior to the cortical thickness in all sections (Figs. 7A and 7B). Cortical thickness is relatively constant, except for the posterior side of the cortex, where periosteal resorption has occurred, and become more pronounced in the distal midshaft (Figs. 7A and 7B). A few isolated trabeculae protrude in the medulla, without forming any conspicuous pattern (Fig. 7C). CCCB is the main type of bone in the inner and mid-cortex (Fig. 7D); it ranges from a loose circular pattern in the innermost cortex (Fig. 7D) to a more irregular vascular pattern, with oblique anastomoses and radial primary osteons (Figs. 7D and 7E). Numerous small secondary osteons are present (Figs. 7E and 7F). In the outer cortex, a layer of woven bone (WB) with small longitudinal and radial canals tops the CCCB on the medial and anterior sides, with a resorption line separating the two (Fig. 7G); in some sections, these small canals are osteons with concentric lamellae, and the WB thus becomes FLB (Fig. 7E). Many large secondary osteons are present locally in the outermost cortex, mostly at the limits between the four main sides (i.e., anterior, posterior, medial, and lateral), but the underlying primary bone matrix is still visible (Figs. 7H and 7I). The outermost cortex consists of a layer of almost avascular PFB, with a highly resorbed periosteal outline, separated from the CCCB (or FLB, depending on the side) layer by a resorption line (Fig. 7J). Extensive periosteal resorption is observed in all sections: the outer PFB layer is at its thickest on the anterolateral side (Fig. 7K), whereas the posteromedial side has a scalloped outline and the PFB layer is almost completely resorbed (Fig. 7L). Growth marks are absent.

## Discussion

### General considerations

The general histological pattern for the midshaft of all six bones is very similar: most of the cortex is comprised of CCCB, with various degrees of Haversian remodeling depending on the considered side of the bone section. An outer layer of periosteal bone, generally comprising PFB, with a few patches of FLB, is present in all bones as well, but is always subject to high periosteal resorption, suggesting a strong cortical drift. Only a few growth marks are visible in this outer cortex, and no External Fundamental System (sensu Huttenlocker, Woodward & Hall, 2013) can be found, despite all of our specimens having reached subadult to adult size. It is thus likely that such signs of cessation of growth, if originally present, were lost through cortical drift or Haversian remodeling (Castanet, 2006).

Cross-sectional shape is variable from one bone to another, but there appears to be similarities between homologous bones in the forelimb and hind limb. The midshaft of the stylopod (humerus, femur) presents a large medullary cavity with a dense trabecular network to support an outward extension formed through periosteal cortical drift (either the deltopectoral tuberosity or the third trochanter), and a thin cortex of even thickness through the section. Conversely, bones in the zeugopod (radius, ulna, tibia, fibula) exhibit a much more compact profile, with a small and ill-defined medulla, and a thick cortex with a very uneven shape. Such a difference in compactness between stylopod and zeugopod has also been documented in deer, which are cursorial mammals (Amson & Kolb, 2016). This profile might reflect stronger biomechanical constraints (Burr & Allen, 2013) on the zeugopod of the aardvark, which is in direct contact with the soil during the power stroke and has to absorb most of the initial shock (Thewissen & Badoux, 1986). In general, the more elliptic shape of the sections of the two zeugopodial bones for both fore- and hind limb, as opposed to the more circular sections of the stylopodial bones, could be associated with a preferential bending direction in their mediolateral axis, thus reinforcing the axial bending resistance of the lower arm and leg during digging, as has been described in many vertebrates (e.g., Currey, 1999; De Margerie et al., 2004; Cubo et al., 2015). However, although the role of the forelimb in the digging process has been well documented in the aardvark (Thewissen & Badoux, 1986; Endo et al., 2002, 2003) that of the hind limb has never been described in the functional context of fossorial behavior. Since the hind limb is involved in pushing the soil back during the burrowing process and does not perform digging *per se* (Melton, 1976; Taylor & Skinner, 2004), it is thus likely to be submitted to more postural rather than behavioral constraints, and any global similarity in microanatomical features between the forelimb and hind limb cannot be unambiguously associated with any biomechanical adaptation.

The periosteal cortex of the femur, tibia, and fibula presents two to four conspicuous resorption lines, and a layer of FLB, which are absent in the bones of the forelimb (only small patches of FLB are present in the radius and ulna). Where not resorbed/remodeled, this periosteal cortex is also generally thicker and more vascularized than in the humerus, radius, and ulna. This might indicate that, even though more resorption lines are present in the hind limb, suggesting more individual resorption events, the outer layer of periosteal bone is slightly better preserved in the hind limb than in the forelimb, which would support the hypothesis of stronger biomechanical constraints on the forelimb during ontogeny, potentially linked with its more prominent role in the digging process (see “Cortical drift and muscle attachment” section below).

### Compacted coarse cancellous bone as main bone tissue type

Compacted coarse cancellous bone was originally described by Enlow (1962a, 1962b) as “a process of endosteal growth in combination with corresponding periosteal resorption” (Enlow, 1962b, p. 276). During this process, coarse cancellous lamellar trabeculae in the medulla of the metaphysis become progressively compacted, and incorporated into the cortex of the diaphysis during longitudinal growth. The original structure of the lamellar trabeculae is still visible after the compaction, which results in a bone that presents a brecciated structure, with abrupt angles between compacted lamellae, explaining its mesh-like appearance under CPL light (Enlow, 1962a, 1962b). CCCB is thus entirely of endosteal origin, and secondary in nature, since compaction is considered a form of remodeling (Enlow, 1963). Formation of CCCB occurs concurrently with that of periosteal bone, and ensures that cortical thickness remains constant through longitudinal growth when strong periosteal cortical drift occurs in the midshaft (Enlow, 1962a, 1962b, 1963, 1968). This process of metaphyseal compaction is common among vertebrates, and CCCB has been documented in the bone microstructure of most major tetrapod clades, for example, non-avian dinosaurs (Chinsamy-Turan, 2005), birds (Biewener & Bertram, 1993), pterosaurs (de Ricqlès et al., 2000; Prondvai et al., 2012), and pseudosuchians (de Ricqlès et al., 2008), but has mainly been reported in mammals, for example, non-human primates (Warshaw, 2008; McFarlin et al., 2008, 2016; Warshaw et al., 2017), humans (Goldman et al., 2009, 2014), and rodents (Enlow, 1963; Kolb et al., 2015; Montoya-Sanhueza & Chinsamy, 2017).

However, finding CCCB in such a large amount in all limb bones of our specimens is surprising, considering CCCB is formed in the early stages of ontogeny and is usually only present, if at all, in much smaller amounts than what we have observed in the aardvark (Montoya-Sanhueza & Chinsamy, 2017). This is because endosteal bone is known to be less resistant to biomechanical constraints than periosteal bone, and although cortical thickening, whether endosteal or periosteal, does increase structural strength and bending rigidity, endosteal thickening is not traditionally considered to be efficient for increasing bone strength (Bertram & Swartz, 1991; Ruff, Holt & Trinkaus, 2006). CCCB itself is considered to be so compliant, when compared to the much denser periosteal bone, that its replacement through extensive Haversian remodeling has been described by Currey (1984, p. 11) as “a classic case of making the best of a bad job.” For this reason, CCCB is usually completely lost during later ontogenetic stages, through endosteal resorption and/or Haversian remodeling, with most of the remaining cortex being produced through periosteal growth; in the limb bones of adult mammals, if present, CCCB only consists of a few small patches in the inner cortex (Sontag, 1986; McFarlin et al., 2008, 2016; Warshaw, 2008; Goldman et al., 2009; Montoya-Sanhueza & Chinsamy, 2017). Bone cortices with a large amount of CCCB have been described in a few mammals, but only in small species of rodents or primates (Enlow, 1962a), some of which live in insular environments associated with very particular ecological constraints (Kolb et al., 2015), or present a process of cortical thickening, with CCCB always associated with a large amount of periosteal bone (Geiger et al., 2013; Montoya-Sanhueza & Chinsamy, 2017). Hence the identification of CCCB as the main bone tissue type in the limbs of fully grown aardvarks, with little to no periosteal bone in the mid- and outer cortex, differs greatly from what would be expected in a medium-sized terrestrial mammal (Kolb et al., 2015). The lack of endosteal resorption and relatively low Haversian remodeling of CCCB in the aardvark could be linked with two main physiological constraints: biomechanical adaptations to fossorial behavior (Thewissen & Badoux, 1986; Endo et al., 2002, 2003, 2012), and/or metabolic adaptations linked with environmental restrictions (McNab, 1979, 1984; Taylor & Skinner, 2004). These two aspects will be discussed below.

### Cortical drift and muscle attachment: the burrowing strategy of the aardvark

To date, no histological study on burrowing mammals has investigated the link between bone microstructure and muscle attachment sites, which can usually be identified in bone histological cross-sections where Sharpey’s fibers are present. Sharpey’s fibers are responsible for the attachment of the fibrous entheses of tendons, ligaments, and the periosteum to the surface of bones (Benjamin et al., 2002), and as such can be functionally interpreted as evidence for muscle attachment points (e.g. Chen et al., 2006). Furthermore, the influence of muscular activity has been shown to have an effect on bone growth and muscle architecture, but not on muscle attachment sites (Rabey et al., 2015). Sharpey’s fibers are generally unaltered by bone resorption and remodeling, and are considered reliable indicators of muscle-bone interactions, even through extensive modifications of superficial bone during ontogeny (Hoyte & Enlow, 1966). For this reason, the strong periosteal resorption experienced by all bones in our specimens do not prevent the functional signal of muscle attachment sites from being investigated. The myological terminology generally follows Voegele (2014), who extensively reviewed the forelimb myology of the aardvark and closely related taxa, and provided a nomenclatural update for many past references on this matter, allowing for a precise comparison between the many different terminologies used in previous studies.

A large number of Sharpey’s fibers have been found in the outer cortex of the radius and ulna, on the posteromedial and anteromedial sides, respectively, where the periosteal bone layer is the thickest for both bones. The radius is located anteriorly to the ulna in the aardvark forearm (Thewissen & Badoux, 1986; Voegele, 2014), and the relative position of both bones does not change during pronation/supination, which is extremely limited in the aardvark (see next paragraph). Hence the two bundles of Sharpey’s fibers in the radius and ulna were facing each other in the living specimens, and correspond to the insertion of one muscle, located in-between the radius and ulna through the whole of the midshaft. This muscle, attached to both bones through a range of interosseous ligaments and membranes, can be identified as the m. pronator quadratus (Thewissen & Badoux, 1986). Humphry (1868) originally described the pronator quadratus as being a small muscle in the aardvark when compared to that of the harbour seal (*Phoca vitulina*). However, it has since been identified as being large and developed in the aardvark compared to that of other large burrowing mammals such as xenarthrans and pangolins, as it occupies the entire interosseous space between the radius and ulna (Galton, 1869b; Sonntag, 1925; Endo et al., 2002; Voegele, 2014). It has a very large tendon, described by Sonntag (1925, p. 395) as being “really an exceedingly powerful ligament” that is “deeply […] connected by ligaments to the radius.” The tendon is also attached to the ulna, carpal and metacarpal bones, and is connected to the corresponding flexor muscles (Sonntag, 1925). Endo et al. (2002) originally described other pro- and supinator muscles, such as the pronator teres and supinator longus, as also being well-developed in the aardvark, and suggested that these muscles support powerful pronator-supinator movements during soil-crushing action. However, as observed by Thewissen & Badoux (1986) from X-ray photographs of the aardvark forelimb, neither pronation nor supination are possible in its forearms, due to all pro- and supinators being either reduced or assigned to different functions, such as flexing the elbow joint (i.e., pronator teres) or extending the fingers. The extension of the fingers is maintained through the power stroke by the continuous extension of all carpal joints, which ensures maximum strength and amplitude (Coombs, 1983; Endo et al., 2003), and provides increased resistance to angular perturbation (Lieber & Ward, 2011).

This extremely developed insertion of the pronator quadratus is thus a good marker of the peculiar burrowing strategy of the aardvark. This muscle does not play any role in pronation, and instead prevents rotation of the radius around the ulna by maintaining a strong connection between the two bones, from their diaphysis to distal epiphysis, through an extensive range of ligaments. This can also be linked to the very constrained, flattened shape of the ulna, and to the main axis of the radius being strongly incurved to increase available interosseous space (Sonntag, 1925). Conversely, the insertion of the biceps brachii and clavodeltoideus on the upper part of the radius, similar to that of other mammals, prevents further extension of the elbow, and helps in flexing the shoulder joint and retracting the forelimb at the end of the power stroke (Coombs, 1983; Thewissen & Badoux, 1986). This absence of rotation in the forearm contrasts with that of xenarthrans, pangolins, and monotremes, where pro- and supinators are notably developed, and the rotation of the forearm plays an important part in the power stroke (Galton, 1869a; Sonntag, 1925; Gambaryan et al., 2015; Olson et al., 2016). In these taxa, the pronator quadratus is either associated with pronation (anteaters), reduced (sloths), or absent (armadillos, pangolins, and monotremes; Thewissen & Badoux, 1986). A high rotational ability of the forearm has also been described in larger burrowers, such as the American badger *Taxidea taxus* (Moore et al., 2013).

This complete lack of movement in the aardvark forelimb during the power stroke thus might reflect highly autapomorphic constraints associated with its extensive digging behavior, possibly linked with its large size for a burrower. The forearm is responsible for most of the initial phase of the power stroke, and must be able to support strong bending forces through (1) increased resistance of the radius and ulna, and (2) flexion of the elbow joint, since such forces cannot be dissipated by flexing the fingers or rotating the wrists. An alternative hypothesis to explain this restriction of movements in the forearm would be a plesiomorphic condition in the clade Afrotheria. Indeed, a lack of pronation or supination in the forelimb, along with reduction in size or absence of associated muscles, has been documented in elephants (Harris, 2010), hyraxes (Reif, Thomas & Fischer, 1985), and golden moles (Gasc et al., 1986), and hypothesized in elephant shrews (Voegele, 2014) and tenrecs (Salton & Sargis, 2008; Voegele, 2014). Such characteristics, however, may have evolved independently in each of those taxa due to other evolutionary constraints, such as graviportality (elephants) or a subterranean lifestyle (golden moles). Similarly, the large insertion of the pronator quadratus observed in the aardvark cannot be found in any other afrothere, and more research on the myology and bone histology of afrotheres is needed to assess a possible plesiomorphic lack of rotation in the forelimb for this clade.

This strategy also reflects on the muscle insertions of the arm, as can be seen in the humeral sections of our specimens. Although we did not identify Sharpey’s fibers in the deltopectoral tuberosity—likely because of the large amount of Haversian remodeling—its highly constrained and protuberant shape, particularly on its medial side, corresponds to the attachment point of the deep portion of the m. pectoralis superficialis (Voegele, 2014), also described as pectoralis major in previous studies (Humphry, 1868; Galton, 1869b). The pectoralis superficialis inserts via a tendon also connected to the biceps brachii, clavodeltoideus, and brachialis (Galton, 1869b; Voegele, 2014). The large insertion of biceps brachii and clavodeltoideus on the radial tuberosity was also observed in a longitudinal section of the proximal radial epiphysis in one of our specimens (L. J. Legendre, 2016, personal observation). Overall, the humerus is a short, strong bone that is the most stable element of the forelimb during the power stroke (Thewissen & Badoux, 1986), as most biomechanical forces apply to the shoulder joint and antebrachium. The power stroke of the forelimb during digging involves the abduction of the forearm, which requires an outward rotation of the humerus that has been described as being operated by the pectoralis superficialis and subscapularis (Thewissen & Badoux, 1986). It is thus not unexpected to observe a deltopectoral tuberosity that is notably large and flattened at its extremity to allow for the insertion of a strong tendon (Le Gros Clark & Sonntag, 1926). An enlarged deltoid or deltopectoral crest is found in many other scratch-digging and hook-and-pull digging mammals (sensu Hildebrand, 1985), including xenarthrans (i.e., armadillos and anteaters) and true moles (Thewissen & Badoux, 1986). However, the pectoralis superficialis is absent in those groups (Galton, 1869a; Olson et al., 2016), and the outward rotation of the humerus is ensured by either the subscapularis, in anteaters and moles, or the spinodeltoideus, in armadillos (Sonntag, 1925; Thewissen & Badoux, 1986). In both armadillos and anteaters, the deltoid tuberosity is thinner and more elongated than that of the aardvark (Sonntag, 1925) and supports the insertion of the deltoid muscles via numerous tendinous fibers. In contrast, deltoid muscles in the aardvark are more reduced and connected to the pectoralis superficialis (Shrivastava, 1962). Voegele (2014) describes the m. acromiodeltoideus and spinodeltoideus as being fused, and inserted via a short tendon to the deltoid ridge. The humerus acts as a strong support for the antebrachium, to dissipate the bending forces through flexion of the elbow joint (Thewissen & Badoux, 1986). Hence the shape of the deltoid ridge in the humerus, extremely flattened through extensive periosteal resorption (Fig. 2A), as well as the extended deltopectoral tuberosity in the lower midshaft (Fig. 2B), might reflect the need for a larger insertion than in other burrowers to support both pectoral and deltoid muscles, involved in maintaining the humerus in an abducted position through the power stroke.

The stronger biomechanical constraints on the lower arm, compared to those on the upper arm, could thus explain both the higher amount of Haversian remodeling and the thicker periosteal bone layer observed in the radius and ulna compared to that of the humerus. An elliptical shape of the medullary cavity has been suggested as resulting from high torsion forces in the humerus of true moles (Meier et al., 2013). Indeed, the initial abduction and axial rotation of the humerus represent the main driving force of the power stroke in that group, and provide stabilization of the elbow and shoulder joints to absorb resistance forces from the walls of the burrow (Rose et al., 2013). In this regard, the elliptical shape of the medulla in the radius and ulna of the aardvark also matches the stronger torsion and bending constraints experienced by these bones compared to the humerus, which has an enlarged and more circular medullary cavity—although the digging style of true moles, described by Hildebrand (1985) as rotational digging, is distinct from that of scratch-diggers like the aardvark. As mentioned above, such constraints could also explain the greater relative cortical thickness observed in the radius and ulna compared to that of the humerus. However, such a high cortical thickness could have been achieved through periosteal cortical thickening (Kolb et al., 2015; Montoya-Sanhueza & Chinsamy, 2017), rather than through the maintenance of CCCB. Similarly, the periosteal cortex could have exhibited denser HB, as can be found in many large mammals (Enlow & Brown, 1958; de Ricqlès et al., 1991). Other factors susceptible to influencing bone microstructure must therefore be considered in addition to this strictly biomechanical and structural interpretation (Martin et al., 2015).

As mentioned previously, the aardvark hind limb presents a general histological pattern very similar to that of the forelimb, that is, the rounder cross-sectional shape, greater cortical thickness, and higher proportion of periosteal bone in the zeugopod than in the stylopod. The outer cortex of the tibia presents a layer of FLB that is thickest on its posterior side, thus facing the thick layer of WB on the anterior side of the fibula; this configuration matches that of the radius and ulna for the insertion of the pronator quadratus. However, no Sharpey’s fibers were observed in the tibia and fibula, and there is no equivalent of the pronator quadratus that inserts in-between those two bones in the aardvark hind limb (Sonntag, 1925). The curved edges of the third trochanter, although not associated with any Sharpey’s fibers, do correspond to the insertion of the gluteus maximus, but a similar insertion zone for this large muscle can be found in most terrestrial mammals (Humphry, 1868; Galton, 1869a, 1869b; Sonntag, 1925). The well-developed pectineal tuberosity also has not been the subject of any hypothesis on a potential role of the pectineus in the digging process—although it might be a plesiomorphic feature in Afrotheria, since this muscle was also documented as being very developed in hyraxes (Murie & Mivart, 1865). Consequently, the lack of previous studies on the biomechanical role of the aardvark hind limb during digging prevents any further functional interpretation of its histological patterns, and the organisational similarities between fore- and hind limb are likely to be influenced by other parameters than just fossorial behavior.

### Metabolic and environmental constraints

As a medium-sized mammal, the aardvark is subject to unique ecological constraints on its metabolic rate by being both a burrower and an anteater (Taylor & Skinner, 2004). The semi-arid environment of the South African Karoo, where our specimens were originally collected, has a temperature range between 35 °C in summer and –15 °C in winter (Taylor & Skinner, 2004). To survive such extreme temperatures, aardvarks tend to be active at night and dig very deep burrows (Platt et al., 2016), which provide an environment with lower gas exchanges, and where the ambient temperature only varies between 5 and 10 °C (Taylor & Skinner, 2004). These low temperatures also prevent them from reaching hyperthermia caused by their digging activity, which produces a lot of body heat—an effect accentuated by their large body mass (McNab, 2002). To maximize the loss of this excess heat during fossorial activity, the aardvark presents a sparse fur coat, ensuring a very high minimal thermal conductance, which is, conversely to that of smaller fossorial mammals, independent of its body mass (McNab, 1979). This process also reduces oxygen consumption and water loss (Reichman & Smith, 1990). Myrmecophagy is also a limiting factor for metabolic activity: ants and termites are hard-to-catch prey with low nutritional value, and when compared to other mammals with different food habits, myrmecophagous mammals in general present the lowest of all mass-independent basal metabolic rates (McNab, 1984, 2008). Hence the basal metabolic rate of an adult aardvark ranges between 0.1 and 0.4 mL O_2_ g^−1^ h^−1^ (McNab, 1984), an extremely low value for a mammal that size (White & Seymour, 2003; Genoud, Isler & Martin, 2018). For this reason, such high metabolic and energetic constraints may be a limiting factor on extensive periosteal cortical thickening and the resorption of CCCB. These constraints might even be stronger than in other burrowing anteaters, since aardvarks start digging their own burrows at six months of age, when they are still actively growing—adult size being reached at about one year of age (Shoshani, Goldman & Thewissen, 1988)—while other large anteaters such as armadillos only start burrowing on their own when close to adult size (McBee & Baker, 1982; McDonough et al., 2000). The large amount of energy required by this digging process during the phase of active growth (Hildebrand, 1985; McNab, 2002) may begin to constrain periosteal bone growth from six months old.

Considering both biomechanical and environmental constraints, it appears that the osteohistology of the aardvark reflects a physiological compromise between these two factors (see a review in Ruff, Holt & Trinkaus, 2006). Due to our small sample and lack of juvenile specimens, deciphering the general histological growth pattern of the aardvark is not possible at this stage, but several hypotheses can nonetheless be proposed to explain the way all these different constraints may act together to shape the limb bone histology in our specimens. In the first six months after they are born (body size ≈ 55–120 cm), juvenile aardvarks do not yet have the physical and energetic constraints linked with daily burrowing and foraging activity (Shoshani, Goldman & Thewissen, 1988); consequently, bone growth rates are likely relatively high (as seen by the presence of remaining unremodeled FLB patches in some elements). During this phase, most of the midshaft is likely to be formed through periosteal growth (as is typical of extant mammals—e.g., Enlow, 1963), while coarse cancellous trabeculae are formed in the metaphysis, through the remodeling of calcified cartilage trabeculae into metaphyseal bone (Martin et al., 2015). At six months of age (body size ≈120 cm; Shoshani, Goldman & Thewissen, 1988), when the animals start displaying intense burrowing activity, the strong bending and torsion constraints associated with fossoriality could in theory induce periosteal cortical thickening to increase bone resistance (Ammann & Rizzoli, 2003; De Margerie et al., 2004, 2005; Montoya-Sanhueza & Chinsamy, 2017)—potentially stimulated by higher muscle activity, which is known to influence bone growth and outer morphology (Currey, 2002; Burr & Allen, 2013; Rabey et al., 2015).

However, as previously shown, metabolic restrictions associated with environmental and diet-related constraints (McNab, 1979, 1984, 2008) might prevent a cortical thickening as high as that observed in the limb bones of true moles or subterranean rodents. This might explain why the maintaining of endosteal bone is favored over periosteal thickening, with a lack of resorption of the previously formed metaphyseal trabeculae after their inclusion in the diaphyseal cortex, resulting in a thick layer of CCCB (Enlow, 1962a, 1962b). Although CCCB has been described as being of reduced strength for impacts and fractures compared to other bone tissue types (Currey, 2002), its compliant structure might actually be an advantage in the context of strong bending constraints. Less stiff bones usually tend to be more compliant (Currey, Pitchford & Baxter, 2007); cancellous bone is known to present a lower mineral content and tissue density, and a higher water content, than cortical bone, which considerably reduces its stiffness (Martin et al., 2015). Increased bone compliance reduces the efficiency of muscular action, but increases the amount of energy a bone can absorb before reaching its failure load (Currey, 2004), and can therefore be an advantage for structures prone to absorb a large amount of bending stress, such as the aardvark forelimbs during the initial stages of the power stroke (Martin et al., 2015). Since cancellous bone requires less energy than cortical bone to carry around due to its lower density (Martin et al., 2015), a high amount of CCCB in the cortex could also be a good way to reduce metabolic energy consumption, which would corroborate the hypothesis of a physiological compromise to address both energetic and structural limitations.

The initial periosteal growth in the aardvark limb bones thus gives way to a high periosteal resorption, resulting in cortical drift, stronger in the zeugopod, reinforcing axial bending resistance, and occurring at the same time as the endosteal thickening through formation of CCCB (Enlow, 1963; Martin et al., 2015). The overall shape of the sections is clearly constrained by the need for large muscle insertions, for example, the large flattened deltopectoral tuberosity in the humerus. Although this tuberosity has been shaped by strong periosteal remodeling, it is important to note that muscle-induced tension can be associated with simultaneous bone deposition or removal, without necessarily implying any modification of the size or shape of the bone surface where the attachment occurs (Rabey et al., 2015). Similarly, the lack of Sharpey’s fibers in the highly remodeled CCCB forming the deltopectoral tuberosity can be explained by the fact that, in such a context of periosteal resorptive destruction, the fibers ensuring the attachment of muscles to the bone surface are not strictly speaking Sharpey’s fibers, but intercellular collagenous fibers—similar to what can be found in all bone tissues—which ensure a direct continuity between muscle and bone surface, and are not disturbed by the resorption process (Hoyte & Enlow, 1966). Thus, the strong humeral periosteal resorption at the level of the deltopectoral tuberosity is not incompatible with the high muscular activity of the associated pectoral muscles attached to it. Conversely, the large amount of periosteal bone at the insertion of the pronator quadratus in the radius and ulna might be linked with the fact that this muscle does not apply any strain constraint during fossorial activity, but instead maintains the two bones together through a range of ligaments, and prevents any rotation of these bones around each other (Thewissen & Badoux, 1986). In that scenario, the radius and ulna would act as one structural unit, which would explain why cortical drift only induces periosteal resorption on the sides opposite to the insertion of the pronator quadratus, leaving the periosteal surface unmodified at the attachment site.

In the later phases of growth, that is, when the animal reaches adult size (≈140–220 cm) the slowing down of bone resorption combined with a larger body size is likely to produce microcracks in the outer cortex, which might explain the high Haversian remodeling occuring in all bones, and becoming gradually denser from the mid- to the outer cortex (Currey, 2003). The CCCB, however, apart from a few patches of dense HB in the ulna and tibia, is never entirely remodeled, contrary to what can be observed in the bones of most other mammals (McFarlin et al., 2008, 2016; Goldman et al., 2009, 2014). Secondary remodeling of CCCB allows the development of a new pattern of collagen fiber orientations to improve strain resistance, but unremodeled CCCB in the limb bones of mammals has been documented, even when the orientation of collagen fibers in CCCB does not match the mechanical strain environment (Bromage & Boyde, 1998; McFarlin et al., 2008; Warshaw et al., 2017). To explain this apparent paradox, McFarlin et al. (2008) and Warshaw et al. (2017) suggested that species- and bone-specific construction rules, as well as physiological constraints, might have a stronger influence on Haversian remodeling in CCCB than previously expected. The influence of local mechanical loading on bone microstructure through ontogeny, as described by the so-called Wolff’s Law (although not originally proposed as such by Wolff—see Kivell, 2016), is indeed only one of many physiological constraints that can influence the orientation of collagen fibers and/or trabeculae (Ruff, Holt & Trinkaus, 2006), and the high metabolic constraints associated with a fossorial lifestyle may thus also have an influence on the organization of secondary osteons that can be locally formed in one particular bone. In our specimens, secondary osteons are most densely packed at the limit between periosteal and endosteal bone, often aligned along the resorption line separating the two. This could also be linked with what has been hypothesized as vascular continuity, that is, a high number of secondary osteons of periosteal origin in endosteal bone when strong periosteal resorption occurs (Enlow, 1962b). These secondary osteons might serve as support for muscle attachment during external resorption (Enlow, 1962b), or increase the number of vascular canals available to increase blood supply when most primary canals in the periosteal bone have been destroyed by resorption (McFarlin et al., 2008). In our specimens, the outer layer of PFB is indeed almost avascular, and the CCCB layer underneath it often presents a local reticular network formed by anastomoses in its outermost part, locally increasing vascular supply. This highly vascularized pattern suggests a primarily physiological, rather than structural, constraint on the number of secondary osteons in the aardvark limb bones. However, the role of secondary osteons in maintaining vascular continuity has been poorly studied, and further research is required to assess the potential importance of this process in resorptive bone surfaces and at the endosteal bone/periosteal bone interface (McFarlin et al., 2008).

Another hypothesis that could explain the maintenance of CCCB through ontogeny in the aardvark is that it may not necessarily be as disorganized as has been previously suggested. Due to its scarcity in the limb bones of adult tetrapods, CCCB has never been the subject of any large-scale study to assess its mode of formation and functional role in strain, torsion, and/or bending resistance, as have been many other bone tissue types of periosteal origin (Castanet et al., 2000; De Margerie, 2002; De Margerie, Cubo & Castanet, 2002; De Margerie et al., 2004, 2005; Stein & Prondvai, 2014; Mitchell & van Heteren, 2016). CCCB in general is often overlooked in reference publications on bone microstructure terminology (Francillon-Vieillot et al., 1990; de Ricqlès et al., 1991). For this reason, all descriptions of CCCB in vertebrate bones always refer to its original description by Enlow (1962a), and describe it as being very compliant and of reduced strength when discussing its functional significance, but no experimental study with a large sample size has ever been performed on any tetrapod to test this hypothesis. In our aardvark specimens, when observed under CPL light, the orientation of endosteal struts formed by compacted trabeculae appears to be highly organized, forming a mesh of perpendicularly-oriented lamellae that generally follow the orientation of the periosteum and the curvature of the whole section, often matching the corresponding pattern of the trabeculae in the medulla. In two studies on the wing bones of pterosaurs, some of which present a high amount of CCCB, along with strong cortical drift and extensive remodeling (de Ricqlès et al., 2000; Prondvai et al., 2012), these CCCB struts were hypothesized to serve as a support structure for pterosaurian thin-walled bones, which have been shown to experience strong bending and torsion forces (Padian & Rayner, 1993; Palmer, 2011). Indeed, in coarse cancellous bone (CCB), trabeculae tend to align with maximum stresses, following compression and tension lines, through strain-induced stimulation of the osteocyte network (Martin et al., 2015), thus corresponding to classical hypotheses on the influence of mechanical forces on trabecular architecture (although the important variability of trabecular bone anisotropy in the skeleton makes any generalization difficult; see Keaveny & Hayes, 1993). This ensures comparative strain equilibrium, reduces shearing stresses, and stimulates bone matrix formation in osteoblasts (Burger & Veldhuijzen, 1993; Martin et al., 2015, and references therein). Similarly, during cortical bone modeling, collagen fibers tend to align in parallel to the bone long axis when submitted to tensile forces, or perpendicularly to it when submitted to compressive forces (Kini & Nandeesh, 2012). CCB consists of trabecular bone with a spatial arrangement matching these preferential orientations to resist either compressive or tensile loads. During compaction of CCB to form cortical CCCB, the deposition of new lamellae within the spaces of cancellous bone is constrained by this preexisting trabecular network, and the lamellae thus follow these orientations as well (Enlow, 1962a, 1968). The resulting mesh-like network of lamellae may thus potentially display increased resistance to both tensile and compressive forces, and could provide stronger biomechanical support during growth than previously expected. Hence, in this hypothesis, periosteal cortical drift in the aardvark bones would only act as a supplementary reinforcement of bending and strain resistance, already partially ensured by the inner organization of CCCB.

## Conclusion

In this study, we described the limb bone histology of the aardvark for the first time, in the context of its fossorial behavior. We identified CCCB as being the main bone tissue type in all limb bones of the aardvark, with periosteal bone being highly resorbed through strong cortical drift. To explain this histological pattern, highly unusual among adult mammals and adult vertebrates in general, we have presented two hypotheses to explain the maintenance of CCCB through ontogeny, both of which involve a physiological compromise between the influence of structural and environmental factors. The first hypothesis suggests that CCCB is not replaced through ontogeny due to strong metabolic constraints linked with food availability and underground environment, and stress and strain resistance in the limbs is achieved through cortical drift and Haversian remodeling. The second hypothesis proposes that CCCB is not as compliant as was previously assessed, and its structure provides part of the stress and strain resistance required when digging; cortical drift is an additional way to increase this resistance, and Haversian remodeling is influenced mostly by physiological constraints on blood vascular supply in the bone.

These two hypotheses are not necessarily mutually exclusive. One main limitation of our histological descriptions and ensuing discussion is a small sample size, and the lack of juvenile specimens to compare limb bone histology through a whole ontogenetic series. The ontogenetic signal present in such specimens might reveal more about the sequence of periosteal bone growth, compaction of trabeculae, and subsequent local remodeling of CCCB. Similarly, the sequence of conflicting physiological constraints during the different phases of bone growth cannot be deduced from our sample, and it is possible that body size has a strong effect on the relative importance of such constraints. CCCB, for example, could very well provide enough bending resistance under a certain size threshold in juveniles, so that our second hypothesis would apply. In such a case, additional cortical drift and remodeling would only occur in later ontogenetic stages, when endosteal bone alone does not provide enough bending resistance for a higher cortical thickness, and the first hypothesis would then become the new physiological strategy. A larger sample including complete ontogenetic series, as well as specimens from different localities across the whole African continent, are needed to further investigate these hypotheses, and decipher the various conflicting factors influencing bone growth patterns in the aardvark.

Montoya-Sanhueza & Chinsamy (2017, p. 25) rightfully advocated for more research to be performed on the role of CCCB in cortical thickening before assessing any potential functional link with fossoriality, highlighting the fact that “neither subterranean nor aquatic lifestyles stimulate directly the formation of CCCB growth.” Indeed, the many factors described in this study as potentially influencing the formation of CCCB may not be interpreted as unambiguous evidence for an influence of fossorial behavior on the bone histology of our specimens. However, a large amount of CCCB associated with high periosteal cortical drift has since been found in the limb bone histology of two other large burrowing mammals, the nine-banded armadillo *Dasypus novemcinctus* (C. Heck, 2017, personal communication), and the American badger *T. taxus* (L. J. Legendre, 2017, personal observation). Straehl et al. (2013) described the histology of *D. novemcinctus* along with other armadillo species, and although they did not report CCCB in their specimens, the “convoluted shape of secondary osteons visible in [their] Fig. 5C” (p. 7) under CPL light looks very similar to the compacted trabeculae found in CCCB. In fact, due to the lack of recent references on comparative bone histology mentioning CCCB as a bone tissue type, it is possible that CCCB might have been misidentified as a peculiar arrangement of secondary osteons in other previous bone histological descriptions, although further research would be needed to investigate this hypothesis.

These observations thus suggest a potential influence of body size on the formation of CCCB in fossorial mammals; quantitative studies on the functional relationship between relative amounts of CCCB in the cortex and patterns of cortical drift (Maggiano et al., 2015, 2016) will likely help to identify more specific constraints associated with this unusual histological profile in the near future. Overall, many microanatomical and histological features could be used to investigate the influence of fossorial behavior on bone microstructure, and many recent quantitative studies have started to focus on such characters in other bone regions than the cortical bone of the midshaft (Amson et al., 2017). Such a preliminary qualitative assessment of this functional link in an emblematic species like the aardvark is thus only relevant in the larger context of comparative studies, to identify the respective influence of ontogenetic, phylogenetic, structural, and environmental signals in the bone microstructure of fossorial mammals.

## Supplemental Information

10.7717/peerj.5216/supp-1 Supplemental Information 1 List of specimens and histological sections depicted in each panel of every histological figure in this paper (i.e. Figs. 2–7). For each column, a given section number corresponds to that of the section of the bone mentioned in the first line of that column, for each corresponding specimen. For sections taken from specimen MVD-M1, for which bones from both forelimbs were sectioned (see Table 1), the left or right element is specified.

## Institutional Abbreviations

MVD-M: Modern Vertebrate Database—Mammals (Karoo Palaeontology Department)
NMBF: National Museum, Bloemfontein, Florisbad Department—National Museum, Bloemfontein, South Africa
KMM: McGregor Museum, Kimberley, Northern Cape, South Africa.

## Additional Information and Declarations

### Data Availability

The following information was supplied regarding data availability: This is a descriptive study on bone histological thin-sections. All histological features described in the text are illustrated in Figs. 2–7. Macroanatomical measurements mentioned in the “Material and Methods” section are provided in Table 1. Material described in the manuscript is stored at the National Museum, Bloemfontein, South Africa.
