# Supplementary material for: Digging the compromise: investigating the link between limb bone histology and fossoriality in the aardvark (Orycteropus afer)
Source: PeerJ. 2018 Jul 11;6:e5216. doi: 10.7717/peerj.5216 (PMC6045922; doi:10.7717/peerj.5216)
Supplement: Supplemental Information 1 — For each column, a given section number corresponds to that of the section of the bone mentioned in the first line of that column, for each corresponding specimen. For sections taken from specimen MVD-M1, for which bones from both forelimbs were sectioned (see Table 1), the left or right element is specified. [file peerj-06-5216-s001.docx]

SUPPLEMENTARY TABLE 1. List of specimens and histological sections depicted in each panel of every histological figure in this paper (i.e. Fig. 2–7). For each column, a given section number corresponds to that of the section of the bone mentioned in the first line of that column, for each corresponding specimen. For sections taken from specimen MVD-M1, for which bones from both forelimbs were sectioned (see Table 1), the left or right element is specified.

| Figure n°  Panel | 2 (Humerus) | 3 (Radius) | 4 (Ulna) | 5 (Femur) | 6 (Tibia) | 7 (Fibula) |
| --- | --- | --- | --- | --- | --- | --- |
| A | MVD-M1, left humerus, section 5 | MMK 7243, section D2 | MMK 7243, section 7 | NMBF 12311, section 8 | MMK 7243, section 8 | MMK 7243, section 4 |
| B | MVD-M1, right humerus, section 6 | MVD-M1, left radius, section 2 | MVD-M1, right ulna, section 9 | MVD-M1, section 4 | MVD-M1, section 9 | MVD-M1, section 5 |
| C | MVD-M1, left humerus, section 4 | MMK 7243, section D3 | NMBF 12311, section 4 | MVD-M1, section 4 | NMBF 12311, section 5 | MMK 7243, section 4 |
| D | MVD-M1, right humerus, section 7 | NMBF 12311, section 4 | MMK 7243, section 8 | NMBF 12311, section 8 | MVD-M1, section 9 | MVD-M1, section 5 |
| E | NMBF 12311, section 5 | MMK 7243, section D6 | NMBF 12311, section 4 | NMBF 12311, section 8 | NMBF 12311, section 3 | NMBF 12311, section 7 |
| F | NMBF 12311, section 5 | MVD-M1, left radius, section 2 | NMBF 12311, section 4 | MVD-M1, section 5 | NMBF 12311, section 3 | NMBF 12311, section 6 |
| G | MMK 7243, section 2 | NMBF 12311, section 1 | MVD-M1, left ulna, section 7 | NMBF 12311, section 8 | NMBF 12311, section 5 | MMK 7243, section 3 |
| H | NMBF 12311, section 3 | MVD-M1, left radius, section 3 | NMBF 12311, section 4 | NMBF 12311, section 8 | NMBF 12311, section 5 | MMK 7243, section 3 |
| I | NMBF 12311, section 3 | MVD-M1, left radius, section 3 | MVD-M1, right ulna, section 9 | NMBF 12311, section 8 | MVD-M1, section 9 | MMK 7243, section 3 |
| J | NMBF 12311, section 3 | NMBF 12311, section 4 | MVD-M1, right ulna, section 9 | NMBF 12311, section 8 | MMK 7243, section 3 | MMK 7243, section 3 |
| K | NMBF 12311, section 5 | NMBF 12311, section 1 | NMBF 12311, section 4 | NMBF 12311, section 8 | NMBF 12311, section 3 | MMK 7243, section 3 |
| L | MMK 7243, section 2 | MVD-M1, right radius, section 9 | - | MVD-M1, section 4 | NMBF 12311, section 3 | MMK 7243, section 3 |
| M | NMBF 12311, section 5 | MVD-M1, right radius, section 9 | - | NMBF 12311, section 8 | NMBF 12311, section 5 | - |
| N | MMK 7243, section 2 | MMK 7243, section C3 | - | - | - | - |
| O | NMBF 12311, section 5 | MVD-M1, right radius, section 9 | - | - | - | - |
| P | NMBF 12311, section 5 | - | - | - | - | - |
